# Roles of astrocytic connexin-43, hemichannels, and gap junctions in oxygen-glucose deprivation/reperfusion injury induced neuroinflammation and the possible regulatory mechanisms of salvianolic acid B and carbenoxolone

**DOI:** 10.1186/s12974-018-1127-3

**Published:** 2018-03-27

**Authors:** Xiang Yin, Liangshu Feng, Di Ma, Ping Yin, Xinyu Wang, Shuai Hou, Yulei Hao, Jingdian Zhang, Meiying Xin, Jiachun Feng

**Affiliations:** Department of Neurology and Neuroscience Center, the First Hospital of Jilin University, Changchun, Jilin Province 130021 People’s Republic of China

**Keywords:** Oxygen-glucose deprivation/reperfusion, Astrocytes, Connexin-43, Microglia, Salvianolic acid B, Carbenoxolone

## Abstract

**Background:**

Glia-mediated neuroinflammation is related to brain injury exacerbation after cerebral ischemia/reperfusion (I/R) injury. Astrocytic hemichannels or gap junctions, which were mainly formed by connexin-43, have been implicated in I/R damage. However, the exact roles of astrocytic hemichannels and gap junction in neuroinflammatory responses induced by I/R injury remain unknown.

**Methods:**

Primary cultured astrocytes were subjected to OGD/R injury, an in vitro model of I/R injury. Salvianolic acid B (SalB) or carbenoxolone (CBX) were applied for those astrocytes. Besides, Cx43 mimetic peptides Gap19 or Gap26 were also applied during OGD/R injury; Cx43 protein levels were determined by western blot and cytoimmunofluorescene staining, hemichannel activities by Ethidium bromide uptake and ATP concentration detection, and gap junction intercellular communication (GJIC) permeability by parachute assay. Further, astrocyte-conditioned medium (ACM) was collected and incubated with microglia. Meanwhile, ATP or apyrase were applied to explore the role of ATP during OGD/R injury. Microglial activation, M1/M2 phenotypes, and M1/M2-related cytokines were detected. Also, microglia-conditioned medium (MEM) was collected and incubated with astrocytes to further investigate its influence on astrocytic hemichannel activity and GJIC permeability. Lastly, effects of ACM and MCM on neuronal viability were detected by flow cytometry.

**Results:**

We found that OGD/R induced abnormally opened hemichannels with increased ATP release and EtBr uptake but reduced GJIC permeability. WB tests showed decreased astrocytic plasma membrane’s Cx43, while showing an increase in cytoplasma. Treating OGD/R-injured microglia with ATP or OGD/R-ACM induced further microglial activation and secondary pro-inflammatory cytokine release, with the M1 phenotype predominating. Conversely, astrocytes incubated with OGD/R-MCM exhibited increased hemichannel opening but reduced GJIC coupling. Both SalB and CBX inhibited abnormal astrocytic hemichannel opening and ATP release and switched the activated microglial phenotype from M1 to M2, thus providing effective neuroprotection. Application of Gap19 or Gap26 showed similar results with CBX. We also found that OGD/R injury caused both plasma membrane p-Cx43(Ser265) and p-Src(Tyr416) significantly upregulated; application of SalB may be inhibiting Src kinase and attenuating Cx43 internalization. Meanwhile, CBX treatment induced obviously downregulation of p-Cx43(Ser368) and p-PKC(Ser729) protein levels in plasma membrane.

**Conclusions:**

We propose a vicious cycle exists between astrocytic hemichannel and microglial activation after OGD/R injury, which would aggravate neuroinflammatory responses and neuronal damage. Astrocytic Cx43, hemichannels, and GJIC play critical roles in OGD/R injury-induced neuroinflammatory responses; treatment differentially targeting astrocytic Cx43, hemichannels, and GJIC may provide novel avenues for therapeutics during cerebral I/R injury.

**Electronic supplementary material:**

The online version of this article (10.1186/s12974-018-1127-3) contains supplementary material, which is available to authorized users.

## Background

Stroke is one of the major causes of death around the world, and most survivors suffer from disabilities [1]. Although rapid post-ischemic reperfusion is critical for treatment, the occurrence of post-perfusion lesions usually exacerbates penumbra injury [2, 3]. Cerebral ischemia/reperfusion (I/R) injury apparently activates astrocytes and microglia, which then release neurotoxic or neuroprotective cytokines that may be the “culprit” underlying penumbral secondary injuries [4, 5].

In the central nervous system (CNS), astrocytes form a functional syncytial network via their gap junctions and play important homeostatic roles. Connexins are main components of gap junction, and the most abundant connexin in the brain is connexin-43 (Cx43) expressed by astrocytes [6]. Connexins are integral membrane proteins, and a *hemichannel* is formed by six connexin monomers in the plasma membrane. Hemichannel interactions allow the exchange of ions and small molecules that underlies gap junction intercellular communication (GJIC) [7].

Many studies have explored the function of Cx43 hemichannels and GJIC during brain ischemia [8–12]. In the brain, GJIC may permit transmission of both energy metabolites and hazardous molecules. Astrocytic GJIC aids neuronal cells more resistant to oxidative stress in primary cocultures and hippocampal slice culture [8, 10]; blocking astrocytic gap junctions increases the susceptibility of cocultured neurons to glutamate cytotoxicity [12]. Otherwise, some studies also showed that inhibiting astrocytic gap junction permeability may restrict the flow of neurotoxic metabolites and avoid neuronal death [13–15]. Therefore, the role of astrocytic GJIC during ischemic injuries still remains unclear. Hemichannels are generally regarded as “pathological pores” for being abnormally open in response to cellular stress [16–19]. Studies have demonstrated that stimulus-induced hemichannel opening may permit the release of neurotoxic substances, such as adenosine triphosphate (ATP) and glutamate [20, 21]. Notably, hemichannels are one of the most important channels for ATP release [22, 23]. Under ischemic conditions, a large amount of ATP is released by those damaged neurons in the ischemic core [2], and it is reasonable to assume that, in the ischemic penumbra, ATP released from the pathologically opened astrocytic hemichannels will induce further damages to neurons.

Moreover, extracellular ATP released from astrocytes is effective in mediating microglial activation [22], triggering tumor necrosis factor-α (TNF-α) and interleukin (IL)-1β secretion from activated microglia, which further aggravate the inflammatory reactions [24–26]. After a stroke, the newly activated microglia can express different phenotypes in which they undergo morphological changes and play pro- or anti-inflammatory roles [27]. In vitro studies have shown that activated microglia can reduce GJIC while increasing astrocytic hemichannel activity; this effect may be due to those pro-inflammatory cytokines, such as TNF-α and IL-1β [28–30]. Thus, abnormally opened astrocytic hemichannels, secondary ATP release, and activated microglia-mediated neuroinflammation may reinforce each other, contributing to aggravation of post-ischemic tissue damage, and astrocytic hemichannels may be important targets in neuroinflammatory processes, and precisely regulating it may provide protection for I/R injury.

Till now, there have been many reagents or drugs regulating the closing of Cx43 channels; carbenoxolone (CBX), quinine, mefloquine, Cx-mimetic peptides Gap26 and Gap27, etc. are among them [31, 32]. CBX, a semi-synthetic derivative of glycyrrhetinic acid, has been widely used as a gap-junction blocker in diverse pathological processes in the brain [33, 34]. Regretfully, little is known about the molecular mechanisms by which CBX act on connexin channels. However, some researchers have indicated these reagents may act through indirect mechanisms as phosphorylation or internalization of Cx subunits [35, 36]; further research still needed to clarify the potential mechanism of CBX’s blocking process for Cx channels.

Remarkably, the channels possess their own regulatory system. A certain number of phosphorylation sites have been found in Cx43’s C-terminal region, and these are regulated by kinases including Src, protein kinase B (PKB), protein kinase C (PKC), mitogen-activated protein kinase (MAPK), and casein kinase 1 (CK1) [37–40]. It is now clear that channel permeability is downregulated by phosphorylation of the Tyr247 and Tyr265 sites by Src, the Ser279 and Ser282 sites by MAPK, and the Ser368 site by PKC [40]. Cx43 phosphorylation at CK1- or PKB-related sites induces structural changes that promote gap junction assembly [39, 40]. Also, Src activation could subsequently lead to MAPK, PKC, and PKB phosphorylation. Dephosphorylation of astrocytic Cx43 in a model of cerebral ischemia has been reported by Li et al. [41]. Further, Naitoh et al. found that cerebral ischemia causes astrocytic Cx43 to undergo both dephosphorylation and phosphorylation at serine and tyrosine residues [42]. Moreover, alterations in the expression or activity of various kinases and phosphatases mediate tissue-level responses to cerebral I/R injury. For example, total in vivo PKC levels and activity are increased early after ischemia [43, 44], and we previously showed that p-Akt contributed to the protection of salvianolic acids against cerebral I/R injury [45]. Src kinases are activated after global ischemia, and Src inhibitor injecting effectively alleviates ischemic injury [46–48]. Further investigations are needed to elucidate how these protein kinases induce connexin’s phosphorylation or dephosphorylation during cerebral ischemia-induced astrocytic uncoupling.

*Salvia miltiorrhiza*, which is called danshen in Mandarin, is commonly applied in traditional Chinese medicine to treat cardiovascular diseases [49]. Salvianolic acid B (SalB, molecular formula: C36H30O16) is the most abundant bioactive hydrophilic compound of *S. miltiorrhizae* and has been assigned as the marker component for the species in the Chinese Pharmacopoeia [50]. SalB can promote high-energy phosphate compound concentrations and mitochondrial membrane potentials in mouse models of cerebral ischemia and reduce intracellular Ca^2+^ concentrations and apoptosis rates in cell-based assays, which suggests its neuroprotective roles [51, 52]. Recently, the salvianolic acids’ putative protein targets have been studied. SalB regulates kinase-related signaling pathways intracellularly, which indicates that SalB interplayed with phosphotyrosine- or phosphoserine/threonine-binding domains [53–56]. Pan et al. showed that SalB prevented microvascular barrier disruption by directly binding Src [57]. Hence, we hypothesized that SalB may affect astrocytic Cx43 and gap junctions by regulating Src kinase and thereby providing neuroprotection.

In summary, we explored changes in astrocytic Cx43 expression, hemichannel and gap junction permeability, observed microglial activation, and the related phenotypic transformations, and further explored the impact of astrocyte-conditioned medium (ACM) on microglial activation and regulation of astrocytic Cx43 hemichannels and GJIC during OGD/R. We also explored the effects of SalB and CBX on the astrocytic expression of Src, PKC, PKB, and the corresponding phosphorylated Cx43 variants after OGD/R injury, which may elucidate these drugs’ regulatory mechanism during I/R injury.

## Methods

### Isolation and culture of mice astrocytes and microglial cells

Astrocytes and microglial cells were obtained from cerebral cortices of 1-day-old C57BL/6 mice as described previously. The experimental protocols were approved by the Experimental Animal Research Ethics Committee of Jilin University. After achieving confluency at about 14 days in vitro, microglia were isolated from mixed glial cultures via shaking on an orbital shaker at 220 rpm for 1 h. The supernatant containing the detached microglial cells was collected and re-seeded for 1 h to allow microglial attachment. After 1 h, the nonadherent cells were removed. Microglia were isolated to allow for further study. On the other hand, incubation of those left glial cultures with a trypsin solution (0.25% trypsin-EDTA diluted 1:2 in DMEM) for 15–25 min resulted in the detachment of an intact layer of cells in one piece with microglial cells remained attached to the bottom of the well; those detached cells were plated and cultured to achieve confluency, and the above methods with mild trypsinization were performed once again. Then, cells were cultured in Dulbecco’s modified Eagle’s medium (DMEM) containing 10% fetal bovine serum (FBS) and 1% penicillin/streptomycin. Purity of astrocytes and microglia was verified by immunofluorescence staining with rabbit anti-glial fibrillary acidic protein (GFAP) (astrocyte specific marker) (Abcam, Cat#ab7260) and rabbit anti-CD11b (microglia-specific marker) (Abcam, Cat#ab128797). Flow cytometry analysis with anti-CD11b-FITC antibody was also performed. Representative results are showed in Additional file 1: Figure S1.

### OGD/R injury to astrocytes and microglia cells

In vitro, oxygen-glucose deprivation/reperfusion (OGD/R) is a classic model of I/R injury [58]. Astrocyte cultures were subjected to four experimental groups: (1) the normal group; (2) the OGD/R group, astrocytes were washed with PBS three times and cultured in DMEM (no glucose) (Gibco, Life Technologies, Cat#11966025). Cells were designed to grow in an incubator with a mixture of 0.1% O_2_, 94.9% N_2_, and 5% CO_2_ (hypoxia) inlet for 2 h. Then, cells were taken out and incubated in DMEM for 48 h in an atmosphere of 95% air and 5% CO2; (3) the OGD/R-SalB (Tianjin Tably Pride Pharmaceutical Co., Ltd., Tianjing, China) group, astrocytes were washed with PBS three times and re-suspended in DMEM (no glucose) containing SalB (20 μg/mL) during OGD/R injury; (4) the carbenoxolone disodium salt (CBX, Abcam, Cambridge, MA, USA, Cat#ab143590) (OGD/R-CBX) group, astrocytes were washed with PBS three times and re-suspended in DMEM (no glucose) containing CBX (10 μM) during OGD/R injury. MTT tests were applied to observe the effects of different concentrations of SalB (Additional file 1: Figure S2A) and CBX (Additional file 1: Figure S2B), and the optimum protection concentration was selected. Microglia cultures were divided into three experimental groups: (1) the normal group; (2) the OGD/R group; (3) the OGD/R-SalB group. Treatment was similar to that of astrocytes.

Further, selective Cx43 hemichannel blocker peptide Gap19 (Lys-Gln-Ile-Glu-Ile-Lys-Phe-Lys/ KQIEIKKFK, Purity = 98.00%, inhibiting Cx43 hemichannel activity but not gap junctional communication) and Cx43 Gap junction blocker peptide Gap26 (Val-Cys-Tyr-Asp-Lys-Ser-Phe-Pro- Ile-Ser-His-Val-Arg/VCYDKSFPISHVR, Purity = 98.03%, corresponding to residues 63–75 of connexin-43, which is a specific gap junction and hemichannel blocker) were purchased from APE_×_BIO (Houston, TX, USA) and used at 100 μM [59, 60]. Besides, a scrambled control peptide was obtained from R&D Systems, Inc. Astrocyte cultures were subjected to four experimental groups: (1) the normal group; (2) the OGD/R group; (3) the OGD/R-Gap19 group; (4) the OGD/R-Gap26 group.

### Preparation of conditioned medium

Astrocytes or microglia were plated onto six-well plates (Becton Dickinson) and cultured in DMEM. To prepare the astrocyte-conditioned medium (ACM) or microglia-conditioned medium (MCM), cells were subjected to OGD(2 h)/R(48 h). The supernatants were collected, centrifuged at 3000×*g* for 3 min, and stored at − 80 °C until use [61].

For experiments, the thawed conditioned media were mixed with an equal volume of fresh DMEM. The ACM or MCM were further applied as “reperfusion medium” to those cells which have been subjected to OGD. In particular, OGD/R-ACM was pre-incubated with apyrase (10 U/mL for 30 min at 30 °C) named as OGD/R + apyrase-ACM; besides, ATP was added in OGD/R-Gap19-ACM at a final concentration of 100 nM, named as OGD/R-Gap19 + ATP-ACM. Meanwhile, these conditioned media were prepared for detecting concentration of ATP and inflammatory-related cytokines.

Apyrase (ATP/ADP-hydrolyzing enzyme, Sigma, kept as a 100× stock solution in PBS, was used at a final concentration of 10 U/mL) [62] and ATP (adenosine 5′-triphosphate, Abcam, ab146525) were dissolved at 10 mM in PBS and used at 100 nM [63]. All drugs were kept at − 20 °C until use.

### Total and subcellular fractionation

Total cellular proteins were extracted from astrocytes using Minute Total Protein Extraction Kits (Invent Biotechnologies, Eden Prairie, MN, USA, SN002). Subcellular protein fractions (plasma membrane and cytosol) were separated using the plasma membrane protein isolation kits (Invent Biotechnologies, Eden Prairie, MN, USA, SM005) according to the manufacturer’s instructions [64].

For plasma membrane protein isolation, all steps were performed at 4 °C. Briefly, cells were lysed in buffer A in a filter cartridge. After centrifugation at 14,000 rpm for 30 s, pellets were re-suspended and centrifuged at 3000 rpm for 1 min. Supernatant was collected and centrifuged at 14,000 rpm for 10 min. The supernatants were then kept as cytosol protein fraction and the pellet as total membrane fraction, which was re-suspended in buffer B and centrifuged at 10,000 rpm for 20 min. The supernatant was then centrifuged again at 14,000 rpm for 30 min, and the pellet was collected as plasma membrane protein fraction for further experiments [65–67].

### Western blot analysis

Protein concentration was evaluated using the BCA method, and 20–40 μg of total extract was separated on SDS polyacrilamide gels, and then the gel-separated proteins were transferred onto polyvinylidene fluoride (PVDF) membranes and probed using the indicated primary antibodies: overnight at 4 °C with Rabbit anti-pan Cx43(1:1000, Cell signaling technology, Cat#3512); Rabbit anti-phospho-Cx43/ GJA1(Ser 368)(1:1000, Abcam,Cat#ab30559); Rabbit anti-phospho-Cx43 (Ser373) (1:1000, Thermo Fisher Scientific, Cat#PA5-64670); Rabbit anti-phospho-Cx43(Ser265)(1:500, Thermo Fisher Scientific, Cat#PA5-37584); Rabbit anti-PKCε (1:200, Santa Cruz Biotechnology, Cat#sc-214); Goat anti-phospho-PKCε (Ser729) (1: 200, Santa Cruz Biotechnology, Cat#sc-12355); Rabbit anti-Akt(PKB) (1:1000, Cell Signaling Technology, Cat#4691); Rabbit anti-phospho-Akt(Thr308) antibody (1:1000, Cell Signaling Technology, Cat#13038); Rabbit anti-Src (1:1000, Cell Signaling Technology, Cat#2108); Rabbit anti-Phospho-Src(Tyr527) (1:1000, Cell Signaling Technology, Cat#2105); Rabbit anti-Phospho-Src(Tyr416) (1:1000, Cell Signaling Technology, Cat#6943); Rabbit anti-GAPDH antibody (1/5000, Abcam, Cat#ab37168); mouse anti-β-actin(1:1000, Abcam, Cat#ab6276); Rabbit anti-Arginase-1 (1:1000, Cell Signaling Technology, Cat#93668). The membranes were washed and then incubated with secondary antibody for 1 h and then analyzed by an Odyssey infrared imaging system (LiCor, USA). Protein bands were quantified using ImageJ software (http://imagej.nih.gov/ij/), and intensity was expressed as relative value of the control.

### Cytoimmunofluorescence staining

Medium was removed after incubation, and cells were fixed with 4% paraformaldehyde. The fixed cells were washed and permeabilized or not with 0.1% Triton X-100 (Sigma). After incubation with 5% bovine serum albumin (Sigma), cells were incubated with Rabbit anti-GFAP antibodies (1:1000, Abcam, Cat#ab7260), rabbit anti-Cx43(1:75, Cell Signaling Technology, Cat#3512) or Rabbit anti-CD11b (1:1000, Abcam, Cat#ab128797) at 4 °C overnight. Then cells were washed, and the secondary antibodies conjugated with Alexa Fluor-488 (1:500; Abcam, Cat#ab150077) were applied. For nucleus labeling, the cells were incubated with DAPI (1:1000; Beyotime, Cat#C1006). Observations were performed using a fluorescent microscope (Leica).

### Ethidium bromide uptake

For dye uptake experiments, astrocytes cultured were washed and then exposed to 0.5 μM ethidium bromide (EtBr) (Abcam, Cat#ab141391) for 10 min at 37 °C. EtBr is impermeable through membrane but can transit through hemichannels and becomes more fluorescent after binding to DNA. After 10 min exposure to EtBr, astrocytes were washed in Hank’s balanced salt solution (HBSS), fixed in 4% paraformaldehyde (PFA) in PBS, and then sections were mounted in fluoromount and imaged by epifluorescence (518 nm excitation and 605 nm emission) using a microscope (DaiphotNikon) associated with image analyzer software (Lucia-Nikon). Background was evaluated on regions devoid of cell bodies. For each experiment, 10 microscopic fields were captured discretionally. Captured images of EtBr uptake were analyzed by counting the number of EtBr-positive cells per field, using ImageJ program (NIH software; http://imagej.nih.gov/ij/). Data were showed as the number of positive-Etd cells per field [68–70].

### Determination of ATP concentration

ATP levels in conditioned medium were determined using a commercial ATP assay kit (Beyotime, China, Cat#S0026) based on the luciferin-luciferase reaction. The chemiluminescence was measured.

### Parachute assay

For acceptor cell preparation, astrocytes were cultured and grouped as previously described. For donor cell preparation, astrocytes were seeded and cultured in 24-well plates. Cells were washed and treated with 25 μM Calcein-AM (Dojindo Laboratories, Japan, Cat#C326) for 30 min in the incubator. Calcein-AM passively diffuses across cell membrane, and the AM group was cleaved by cellular esterases, allowing fluorescence development, and the resulting polyanionic calcein is thus trapped inside these donor cells. Cells were then detached by 0.25% trypsin (Gibco) and resuspended in DMEM with a concentration of 500 cells/mL. Donor astrocytes were parachuted on “acceptor” astrocytes at a ratio of 1:500. Cells were continually cultured in the incubator for 4 h to attach the culture surface and form gap junction. Flow cytometry was conducted to measure calcein positivity. Calcein^+^ cells that were between negative control I-acceptor cells only, negative control II-acceptor cells together with donor cells with CBX treatment (25 μM) [71] during parachuting and attaching process, and positive control-only donor cells on the fluorescence intensity scale were selected. FlowJo software were used for analysis of data (Tree Star Inc., OR, USA) [72–76].

### Flow cytometry detection for microglial M1/M2 phenotype

Cells were blocked with FcR blocking reagent (BD Biosciences) at 4 °C for 10 mins. Cells were permeabilized with Cytofix/Cytoperm (BD Biosciences) for 25 min on ice followed by staining with Rat Anti-Mouse CD11b-FITC (BD Biosciences, 1:100, Cat#557396), Rat Anti-Mouse CD40-PE(BD Biosciences, 1:100, Cat#561846), and Rat Anti-Mouse CD206-PE (eBioscience™, Cat#12-2061-80) antibodies for 30 min at 4 °C. Cells were then analyzed on a FACS Calibur. Data were analyzed using FlowJo software.

### Cytometric bead array

Cytokines in conditioned medium were measured using a cytometric bead array (CBA) mouse Th1/Th2/Th17 Cytokine Kit (BD Biosciences, Cat#560485), and IL-4, IL-6, IL-10, TNF-α, and IFN-γ were selected as the representative cytokines for M1 and M2 microglia. For supernatants, the total protein concentration was measured using a BCA protein assay kit (Thermo Scientific). The concentrations of the cytokines were normalized to the total protein concentration [27].

### Apoptosis

HT-22 murine hippocampal neuronal cells were resuscitated from cryopreserved cells stored in a laboratory. HT-22 cells were maintained in DMEM, which was supplemented with 10% FBS, 2 mM glutamine, and penicillin/streptomycin. Cells were kept at 37 °C and 5% CO_2_ and passed twice a week with 0.125% trypsin.

Cell apoptosis were induced by OGD 12 h and reperfusion with ACM or MCM for 48 h. HT-22 cells were subjected to OGD for 12 h, then cells were reperfused with astrocyte-conditioned medium and divided into five groups: vehicle group, normal (ACM) group, OGD/R(ACM) group, OGD/R-SalB(ACM) group, OGD/R-CBX(ACM) group. Meanwhile, HT-22 cells were subjected to OGD for 12 h, then cells were reperfused with microglia-conditioned medium and divided into four groups: vehicle group, normal (MCM) group, OGD/R(MCM) group, OGD/R + SalB(MCM) group. Those groups were then designed to be cultured for 48 h. Also, HT-22 cells were subjected to OGD for 12 h, then cells were reperfused with ACM and divided into eight groups: vehicle group, normal (ACM) group, normal+ATP(ACM) group, OGD/R(ACM) group, OGD/R + apyrase(ACM) group, OGD/R-Gap19(ACM) group, OGD/R-Gap19 + ATP(ACM) group, OGD/R-Gap26(ACM) group. Then apoptosis was determined using FITC-Annexin V/PE Apoptosis Detection Kit (BD Biosciences, Cat#556570) according to the manufacturer’s instructions and analyzed by flow cytometer. Tests were repeated in triplicate.

### Statistical analysis

Statistical analysis was performed using SPSS version 23.0 software. Analysis of variance (ANOVA), and post hoc Duncan’s test and Dunnett’s test were used to assess differences among multiple groups. *p* < 0.05 was considered statistically significant. Data are displayed as mean ± standard deviation (SD).

## Results

### Effects of SalB or CBX on Cx43 expression in different subcellular fractions of mouse astrocytes after OGD/R injury

**Fig. 1:**
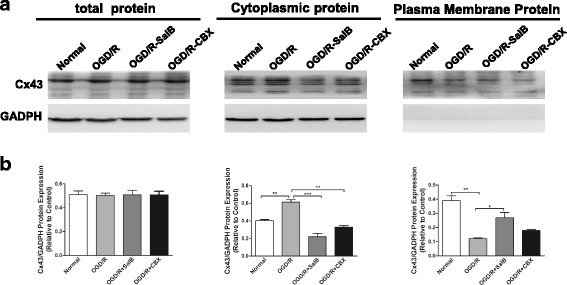
Evaluating the purity of primary cultured astrocytes and Cx43 expression in astrocytic subcellular components. Primary astrocytes were prepared from newborn mice. **a** Western blotting analyses of Cx43 levels were conducted with samples prepared from astrocytes from the control, OGD/R, OGD/R-SalB, and OGD/R-CBX groups. Representative protein bands are displayed for total protein extractions and the plasma membrane and cytoplasm compartments. **b** After OGD/R injury, Cx43 levels were significantly upregulated in the cytoplasm but downregulated in the plasma membrane. SalB treatment reversed these effects. The four groups did not significantly differ in total Cx43 levels. We evaluated the statistical significance with ANOVA and Duncan’s multiple comparisons test. *p < 0.05, **p < 0.01, and ***p < 0.001. Scale bar = 50 μm

We extracted total cellular proteins from cultured astrocytes and conducted western blotting to semi-quantitatively measure Cx43 levels. The four groups did not significantly differ in their Cx43 levels (Fig. 1). We also extracted and isolated proteins specifically from the plasma membrane and cytosolic compartments with a commercial kit. The cytoplasmic Cx43 levels were significantly greater in the OGD/R group than in the normal group (0.612 ± 0.0295 vs 0.403 ± 0.0122, *p <* 0.01), but this elevation was significantly reversed in the OGD/R-SalB (0.219 ± 0.036 vs 0.612 ± 0.0295, *p <* 0.001) and OGD/R-CBX groups (0.329 ± 0.019 vs 0.612 ± 0.0295, *p <* 0.01), compared with that in OGD/R groups. Plasma membrane Cx43 levels were significantly lower in the OGD/R group than in the normal group (0.121 ± 0.0056 vs 0.390 ± 0.0328, *p* < 0.01), SalB treatment increased plasma membrane’s Cx43 compared with that in OGD/R groups, with *p* values < 0.05.

**Fig. 2:**
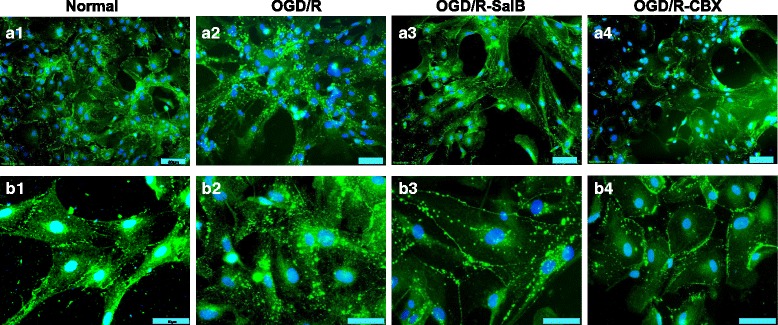
Redistribution of astrocytic Cx43 after OGD/R injury and the effects of SalB and CBX. We cultured primary astrocytes and performed cytoimmunofluorescent staining for Cx43. **a1** In the normal group, Cx43 was mainly expressed discontinuously in the plasma membrane. **b1** At high magnification, Cx43 mainly expressed at the gap junction and some were punctate distributed. **a2, b2** In the OGD/R group, Cx43 was mainly expressed in the cytoplasm, which existed in the shape of block and grain. **a3, b3** Compared to the OGD/R group, the OGD/R-SalB group exhibited weaker cytoplasmic Cx43 staining but enhanced plasma membrane Cx43 staining. The Cx43 expressed at gap junctions was morphologically similar to that in the normal group. **a4, b4** The OGD/R-CBX group exhibited staining results similar to those of the OGD/R-SalB group, though the Cx43 at gap junctions covered a larger area than in the control and OGD/R-SalB groups. Scale bar = 50 μm

Immunocytofluorescence analysis of astrocytic Cx43 expression in the normal group showed that Cx43 was mainly expressed discontinuously in plasma membrane and some in the cytoplasm (Fig. 2, a1). At high magnification, Cx43 was mainly expressed in gap junctions; also, there was some punctate distribution elsewhere (Fig. 2, b1). In the OGD/R group, Cx43 was mainly expressed in the cytoplasm (Fig. 2, a2), with high magnification revealing patchy distributions (Fig. 2, b2). Cytoplasmic Cx43 staining in the OGD/R + SalB group was weaker than that of the OGD/R group but still stronger than that of the control group. Specifically, the OGD/R + SalB group’s gap junction Cx43 expression was similar to that of the control group, but the OGD/R + SalB group’s plasma membrane Cx43 staining was stronger (Fig. 2, a3 and b3). The OGD/R + CBX group exhibited similar staining results, though the gap junction Cx43 distributions typically covered a larger area than in the control and OGD/R + SalB groups (Fig. 2, a4 and b4).

### Effects of SalB or CBX on astrocytic GJIC permeability and hemichannel activity after OGD/R injury

**Fig. 3:**
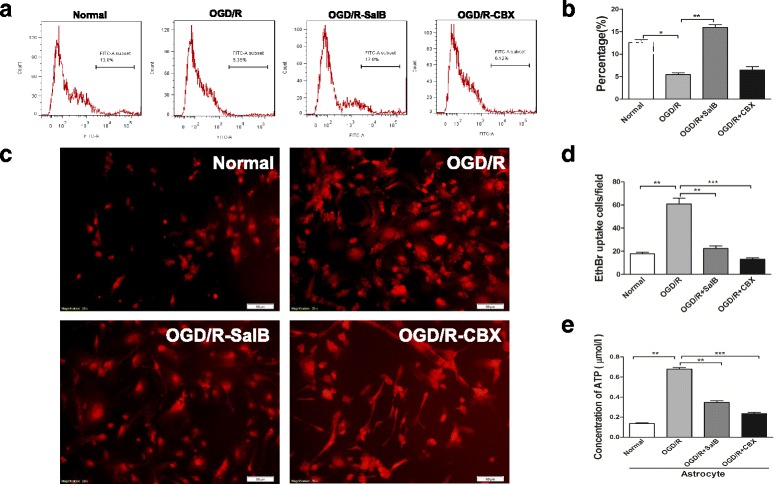
Evaluation of astrocytic GJIC permeability and hemichannel activity after OGD/R injury with SalB or CBX. **a** For GJIC detection, we measured calcein-AM transfer between “donor cells” and “acceptor cells” with flow cytometry. Shown here is a representative flow cytometry plot of transfer after donor astrocytes were labeled with calcein-AM and cocultured with acceptor astrocytes for 4 h. Grouped dye transfer data are shown in **b**. OGD/R injury decreased the degree of astrocytic coupling, but SalB reversed this effect. CBX further inhibited cellular coupling. **c** Representative images depicting ethidium uptake via hemichannels in the four groups. **d** OGD/R injury increased astrocytic ethidium uptake, but SalB and CBX achieved near-significant attenuation of this effect. **e** The supernatant ATP concentration was strongly elevated in the OGD/R group astrocytes, but SalB and CBX reversed this effect. We evaluated the statistical significance with ANOVA and Duncan’s multiple comparisons test. *p < 0.05, **p < 0.01, and ***p < 0.001. Scale bar = 50 μm

As previously mentioned, astrocytic plasma membrane Cx43 proteins form hemichannels that connect the intra- and extracellular compartments, and two opposing channels comprise a gap junction that permits passive intercellular diffusion of small molecules [38]. Herein, we used flow cytometry with cell-permeable fluorescent dye calcein-AM to detect cell coupling. Baseline level of astrocytic gap junction intercellular communication (GJIC) was determined with those astrocytes cultured from control groups. At the same time, the negative control was set by CBX (25 μM) application, which blocked gap junctional communication among astrocytes; the blank control was also set with only acceptor cells detected. The OGD/R group exhibited less calcein-AM-positive cells (%), which were used for indicators of astrocytic gap junction coupling, than the normal group did (5.437 ± 0.418 vs 12.57 ± 0.612; *p* < 0.01). SalB treatment enhanced intercellular dye transfer (15.93 ± 0.601, *p <* 0.01), but CBX treatment inhibited cellular coupling (6.437 ± 0.672) (Fig. 3a, b).

Furthermore, we measured the cultured astrocytes’ EtBr uptake levels, which are considered a functional index of hemichannel activity [64]. The OGD/R group’s astrocytes exhibited significantly greater EtBr uptake than the control group’s astrocytes did (*p* < 0.01). Moreover, the OGD/R-SalB and OGD/R-CBX groups both exhibited significantly weaker EtBr uptake than the OGD/R group did (*p* < 0.01 and *p* < 0.001; Fig. 3c, d).

We used bioluminescence to measure astrocytic ATP release, and the fluorescence levels from five serial ATP dilutions are shown in Additional file 1: Figure S3. The OGD/R group’s astrocyte supernatant ATP concentrations were significantly greater than those of the normal groups (0.680 ± 0.015 vs 0.135 ± 0.014, *p* < 0.01), but the OGD/R-SalB (0.347 ± 0.017) and OGD/R-CBX (0.235 ± 0.013) groups exhibited significant reversal of this elevation (*p* < 0.01 and *p* < 0.01; Fig. 3e).

### Effects of SalB and ACM on microglial activation after OGD/R injury

**Fig. 4:**
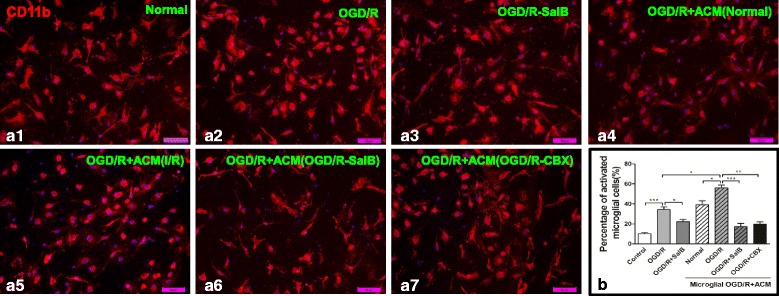
Morphological evaluation of microglia. We separated microglia with a rotary shaker set at 200 rpm for 1 h. **a1**–**a7** OGD/R injury induced microglial activation, as indicated by microglial proliferation and morphological alterations characterized by enlarged, amoeboid somata with short and rare ramifications. SalB attenuated these effects. Further, microglial activation was strengthened by “reperfusion” with ACM from OGD/R group astrocytes, but this effect was reversed when the ACM came from OGD/R-SalB or OGD/R-CBX group astrocytes. Grouped cells’ count data are shown in **b**. We evaluated the statistical significance with ANOVA and Duncan’s multiple comparisons test. *p < 0.05, **p < 0.01, and ***p < 0.001. Scale bar = 50 μm

Microglia, as the CNS’s resident immune cells, constantly monitor for signs of injury. Under resting conditions, they present as ramified morphology. After a stroke, the newly activated microglia can switch to either the M1-pro-inflammatory phenotype with an amoeboid morphology, or the M2 phenotype, in which they perform important roles in restricting inflammation [27]. We separated microglia with a rotary shaker and grew the isolated cells in complete DMEM medium. The OGD/R group exhibited obvious microglial activation, as indicated by numerical proliferation and morphological changes characterized by enlarged, amoeboid somata with short and rare ramifications. SalB treatment attenuated the injury-induced microglial activation (Fig. 4, a1–a3, b; *p* < 0.05). We further examined the effect of ACM on microglial activation. Astrocytes were cultured and grouped as the normal, the OGD/R, OGD/R-SalB, and OGD/R-CBX groups. After OGD(2 h)/R(48 h), we collected supernatants to generate Normal-ACM, OGD/R-ACM, OGD/R-SalB-ACM, and OGD/R-CBX-ACM, respectively, and cultured OGD-treated microglia in them. We found that OGD/R-ACM further activated the microglia but that OGD/R-SalB-ACM and OGD/R-CBX-ACM suppressed microglial activation (Fig. 4, a4–a7, b; *p* < 0.05).

**Fig. 5:**
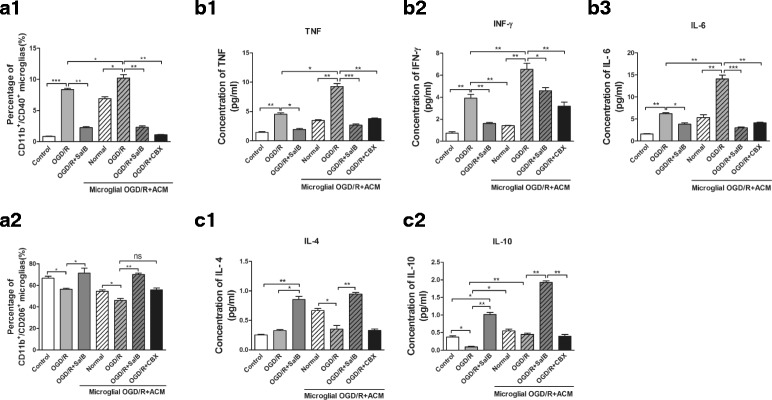
Flow cytometry-based evaluation of microglial subtype polarization and concentrations of M1-related pro-inflammatory and M2-related anti-inflammatory cytokines in cultured microglia supernatants. **a1, a2** We used flow cytometry to evaluate the expression levels of CD40 and CD206, the markers for M1 and M2 phenotypes, respectively. OGD/R injury increased the percentage of CD40^+^CD11b^+^ microglia while decreasing the percentage of CD206^+^CD11b^+^ microglia. SalB reversed these effects. Effect of ACM on microglial polarization was also detected. ACM from OGD/R group significantly increased the percentage of CD40^+^CD11b^+^ microglia, while decreasing the percentage of CD206^+^CD11b^+^ microglia; OGD/R-SalB application decreased the percentage of CD40^+^CD11b^+^ microglia, while it enhanced the percentage of CD206^+^CD11b^+^ microglia; OGD/R-CBX treatment decreased both the percentage of CD40^+^CD11b^+^ and CD206^+^CD11b^+^ microglia; **b**(**1**-**3**), **c**(**1**-**2**) The OGD/R group exhibited increased levels of the M1-related-pro-inflammatory cytokines TNF-α (**b1**), IFN-γ (**b2**), and IL-6 (**b3**), whereas the OGD/R-SalB group exhibited reduced levels of these pro-inflammatory cytokines while increasing the levels of the anti-inflammatory cytokines IL-4 (**c1**) and IL-10 (**c2**). Also, the effects of ACM on M1- or M2-related cytokines were evaluated. We evaluated the statistical significance with ANOVA and Duncan’s multiple comparisons test. *p < 0.05, **p < 0.01, and ***p < 0.001

To determine microglial phenotypes induced by OGD/R injury, flow cytometry was applied to measure the expression of CD40 and CD206, which are markers for the M1 and M2 phenotypes, respectively. We found that the OGD/R group exhibited an elevated percentage of CD40^+^CD11b^+^ microglia (*p* < 0.001) and a decreased percentage of CD206^+^CD11b^+^ microglia (*p* < 0.05). SalB treatment significantly reversed these changes (*p <* 0.05). Moreover, we assayed the ACM’s effects on microglial polarization, and the results are shown in Fig. 5 (a1–a2). OGD/R-ACM induced elevated percentage of CD11b^+^CD40^+^ microglia (*p <* 0.05), while decreased percentage of CD11b^+^CD206^+^ microglia (*p <* 0.05); OGD/R-SalB-ACM inhibited M1 subtype microglia-CD11b^+^CD40^+^ microglia and prompted M2 subtype transformation-CD11b^+^CD206^+^ microglia (*p <* 0.01). For OGD/R-CBX-ACM, both CD11b^+^CD40^+^ and CD11b^+^CD206^+^ microglia were decreased compared with the OGD/R ACM group. We also found that arginase-1 expression was deduced in OGD/R-ACM-treated microglia while elevated in microglia treated OGD/R-SalB-ACM and OGD/R-CBX-ACM (*p <* 0.01) (Additional file 1: Figure S4). The only discrepancy came from the changes of percentage of CD11b^+^CD206^+^ microglia which were decreased after OGD/R-CBX-ACM application while another M2 subtype marker Arginase-I and its protein levels were elevated, which still need further exploration.

We also used flow cytometry with a CBA kit to measure concentrations of M1 phenotype-related pro-inflammatory cytokines (i.e., TNF-α, IFN-γ and IL-6) and M2 phenotype-related anti-inflammatory cytokines (i.e., IL-4 and IL-10) in cultured cell supernatants. The OGD/R group exhibited significantly increased pro-inflammatory cytokine concentrations, whereas the OGD/R-SalB group exhibited reduced pro-inflammatory cytokine concentrations and increased anti-inflammatory cytokine concentrations (*p <* 0.01). The ACM-treated microglia exhibited differential results. OGD/R- ACM treatment significantly induced elevation of concentration of TNF-α, IFN-γ, and IL-6 while it decreased concentration of IL-4 and IL-10. In comparison with OGD/R-ACM group, OGD/R-SalB ACM treatment reversed the effect; OGD/R-CBX ACM application resulted in an apparent decrease of these cytokines, and these results were inconsistent with the phenotype analysis (*p* < 0.01) (Fig. 5, b, c).

### Effects of microglia-conditioned medium (MCM) on astrocytic GJIC permeability and hemichannel activity after OGD/R injury

**Fig. 6:**
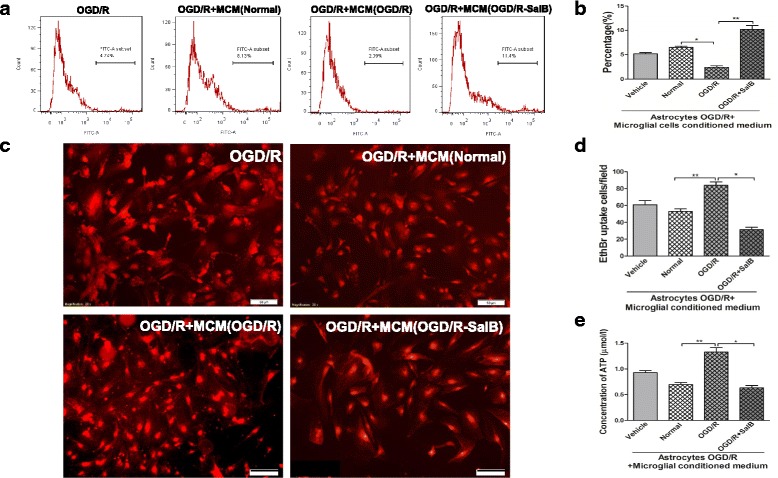
Evaluation of impact of MCM on astrocytic GJIC permeability and hemichannel activity after OGD/R injury. **a** For GJIC detection, we measured calcein-AM transfer between “donor cells” and “acceptor cells” with flow cytometry. Shown here is a representative flow cytometry plot of transfer after donor astrocytes were labeled with calcein-AM and co-cultured with acceptor astrocytes for 4 h. Grouped dye transfer data are shown in **b**. OGD/R-MCM decreased the degree of astrocytic coupling, but OGD/R-SalB-MCM increased astrocytic intercellular dye transfer. **c** Representative images depicting ethidium uptake via hemichannels in the four groups. **d** OGD/R-MCM increased astrocytic ethidium uptake, but this effect was reversed in OGD/R-SalB-MCM-treated astrocytes. **e** OGD/R-MCM increased ATP concentrations in the astrocytic supernatant, but this effect was reversed by OGD/R-SalB-MCM. We evaluated the statistical significance with ANOVA and Duncan’s multiple comparisons test. *p < 0.05, **p < 0.01, and ***p < 0.001. Scale bar = 50 μm

Microglia were separated and subjected to OGD/R injury with or without SalB. After 48 h, we collected the MCM supernatants (i.e., OGD/R-MCM and OGD/R-SalB-MCM) for culturing OGD/R group astrocytes. Compared to the vehicle-treated OGD/R group’s astrocytes, the OGD/R-MCM-treated OGD/R group astrocytes exhibited weakened cellular dye coupling but significantly increased EtBr uptake (*p* < 0.05) (Fig. 6a, b). Moreover, the OGD/R-SalB-MCM induced significant reduction of astrocytic EtBr uptake (*p* < 0.01) and enhanced cell dye transfer levels relative to the OGD/R-MCM (*p <* 0.01) (Fig. 6c, d). We also found elevated ATP concentrations in the supernatant from OGD/R-MCM-treated astrocytes, but this effect was significantly reversed in the supernatants from OGD/R-SalB-MCM-treated astrocytes (*p <* 0.05, Fig. 6e).

### Effects of ACM and MCM on HT-22 neuronal cell lines after OGD/R injury

**Fig. 7:**
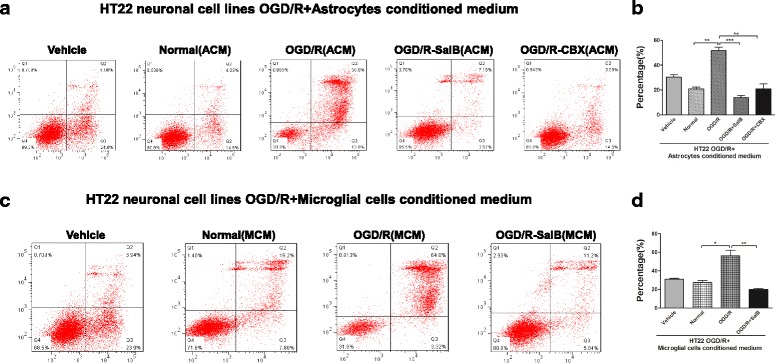
Effects of ACM and MCM on HT-22 neuronal cell lines subjected to OGD/R injury. Cell apoptosis rates in HT-22 murine hippocampal cells, as measured by flow cytometry with an AnnexinV-FITC/PI Apoptosis Detection Kit. **a, b** The OGD/R group HT-22 cells exhibited higher apoptosis after reperfusion with OGD/R-ACM than after reperfusion with Normal-ACM (51.78 ± 4.66% vs. 20.81 ± 2.65%, *p <* 0.01). This increase was reversed in HT-22 cells treated with OGD/R-SalB-ACM (13.86 ± 2.90%, *p <* 0.001) or OGD/R-CBX-ACM (16.98 ± 3.96%, *p <* 0.01). **c, d** MCM had an effect similar to that of ACM. We evaluated the statistical significance with ANOVA and Duncan’s multiple comparisons test. *p < 0.05, **p < 0.01, and ***p < 0.001

To explore ACM’s effects on neuronal survival, HT-22 murine hippocampal neuronal cells were cultured and subjected to OGD for 12 h, then ACM were reperfused and cell viability was examined after a 48-h incubation period. We conducted flow cytometry analysis with an Annexin V-FITC/PI Apoptosis Detection Kit and found that the OGD/R-ACM-treated neurons exhibited a higher apoptosis rate than the untreated neurons did (51.78 ± 4.66% vs 20.81 ± 2.65%, *p <* 0.01). This increase was reversed in neurons treated with OGD/R-SalB-ACM (13.86 ± 2.90%, *p* < 0.001) or OGD/R-CBX-ACM (16.98 ± 3.96%, *p <* 0.01)(Fig. 7a, b). We obtained similar protective effects of OGD/R-SalB-MEM for HT-22 neurons after OGD/R injury (Fig. 7c, d).

### Effects of Gap19 or Gap26 on astrocytic GJIC permeability and hemichannel activity after OGD/R injury

**Fig. 8:**
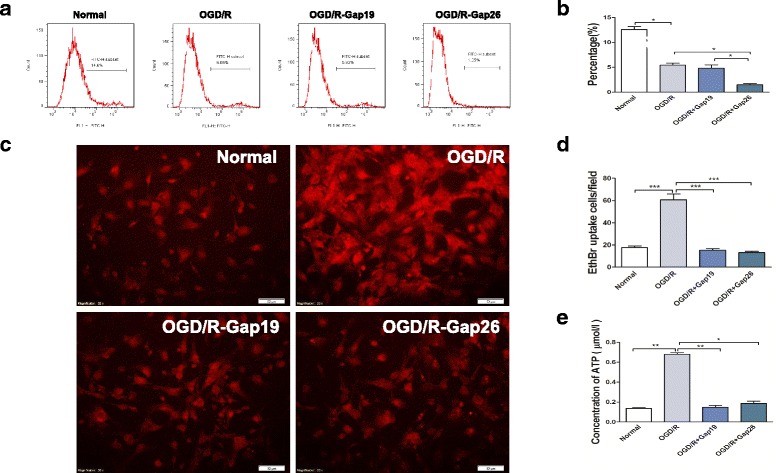
Evaluation of astrocytic GJIC permeability and hemichannel activity after OGD/R injury with Gap19 or Gap26. **a** For GJIC detection, we measured calcein-AM transfer between “donor cells” and “acceptor cells” with flow cytometry. Shown here is a representative flow cytometry plot of transfer after donor astrocytes were labeled with calcein-AM and cocultured with acceptor astrocytes for 4 h. Grouped dye transfer data are shown in **b**. OGD/R injury decreased the degree of astrocytic coupling; Gap26 further inhibited cellular coupling. **c** Representative images depicting ethidium uptake via hemichannels in the four groups. **d** OGD/R injury increased astrocytic ethidium uptake, while Gap19 and Gap26 achieved similar attenuation of hemichannel opening. **e** The supernatant ATP concentration was strongly elevated in the OGD/R group astrocytes, but Gap19 and Gap26 reversed this effect. We evaluated the statistical significance with ANOVA and Duncan’s multiple comparisons test. *p < 0.05, **p < 0.01, and ***p < 0.001. Scale bar = 50 μm

Considering that neither SalB nor CBX is a Cx43 hemichannel or gap junction-specific blocker, we further applied specific Cx43 mimetic peptides Gap19 and Gap26 to conduct related research, so as to clarify its accurate role during OGD/R injury. As previously mentioned, we used flow cytometry with cell-permeable fluorescent dye calcein-AM to detect cell coupling. A baseline level of astrocytic gap junction intercellular communication (GJIC) was determined with those astrocytes cultured from control groups. The OGD/R group exhibited less calcein-AM-positive cells (%). Gap26 treatment further inhibited cellular coupling (1.450 ± 0.225, *p* < 0.05) (Fig. 8a, b).

Furthermore, we measured the cultured astrocytes’ EtBr uptake levels. The OGD/R group astrocytes exhibited significantly greater EtBr uptake than the control group’s astrocytes did (*p <* 0.001). Moreover, the OGD/R-Gap19 and OGD/R-Gap26 groups both exhibited significantly weaker EtBr uptake and ATP release than the OGD/R group did (*p <* 0.001; Fig. 8c, d). For ATP release detection, the OGD/R-Gap19 (0.147 ± 0.034) and OGD/R-Gap26 (0.185 ± 0.024) groups exhibited significant reversal, compared with that in OGD/R group (*p <* 0.01 and *p <* 0.05; Fig. 8e).

### Effects of hemichannel inhibitor or ATP on microglial activation after OGD/R injury

**Fig. 9:**
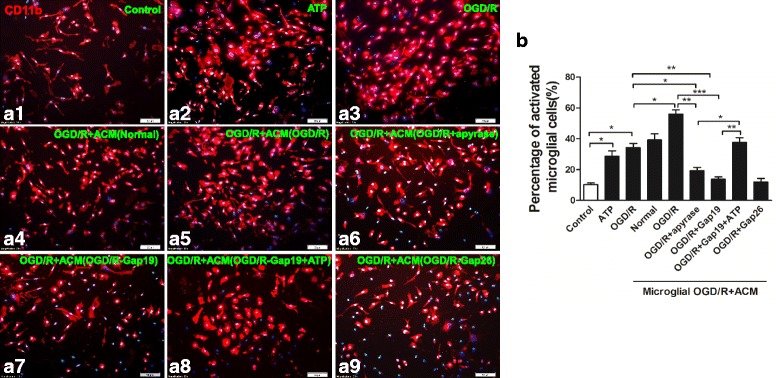
Morphological evaluation of microglia. We separated microglia with a rotary shaker set at 200 rpm for 1 h. **a1**–**a9** Both ATP application or OGD/R injury induced significant microglial activation, as indicated by microglial proliferation and morphological alterations characterized by enlarged, amoeboid somata with short and rare ramifications. Further, microglial activation was strengthened by “reperfusion” with ACM from OGD/R group astrocytes, but this effect was reversed when the ACM came from OGD/R + Gap19 or OGD/R + Gap26 group astrocytes; Further, OGD/R-ACM incubated with apyrase decreased percentage of activated microglial cells, while OGD/R-Gap19-ACM containing ATP-enhanced microglial activation. Grouped cells’ count data are shown in **b**. We evaluated the statistical significance with ANOVA and Duncan’s multiple comparisons test. *p < 0.05, **p < 0.01, and ***p < 0.001. Scale bar = 50 μm

We separated microglia with a rotary shaker and grew the isolated cells in complete DMEM. The OGD/R group exhibited obvious microglial activation, as previously described. Both ATP treatment and OGD/R injury enhanced microglial activation (Fig. 9, a1–a3, b; *p* < 0.05). We further examined the effect of ACM on microglial activation. Astrocytes were cultured and sub-grouped as described previously. After 48 h, we collected supernatants and cultured OGD-treated microglia in them. We found that OGD/R-ACM further activated the microglia, but that OGD/R-Gap19-ACM, OGD/R-Gap26-ACM, and OGD/R + apyrase suppressed microglial activation. On the other hand, OGD/R-Gap19 + ATP-ACM enhanced microglial activation (Fig. 9, a4–a9, b; *p <* 0.05).

**Fig. 10:**
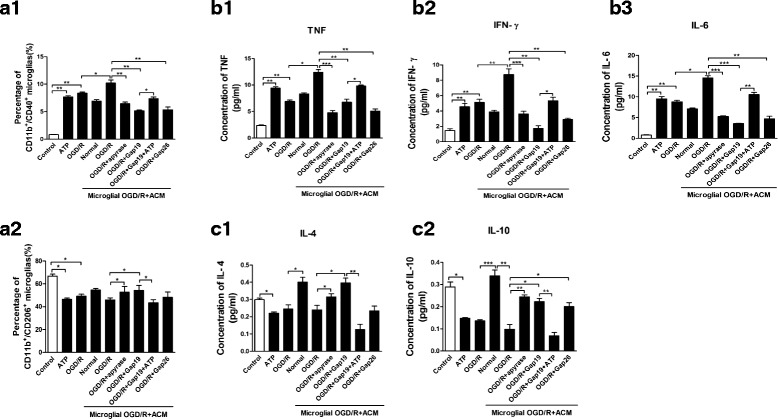
Flow cytometry-based evaluation of microglial subtype polarization and concentrations of M1-related pro-inflammatory and M2-related anti-inflammatory cytokines in cultured microglia supernatants. **a1**, **a2** We used flow cytometry to evaluate the expression levels of CD40 and CD206, the markers for the M1 and M2 phenotypes, respectively. OGD/R injury or ATP application under normal conditions increased the percentage of CD40^+^CD11b^+^ microglia while decreased the percentage of CD206^+^CD11b^+^ microglia. Effect of ACM on microglial polarization was also detected. ACM from OGD/R group significantly increased percentage of CD40^+^CD11b^+^ microglia, while decreased percentage of CD206^+^CD11b^+^ microglia; OGD/R + Gap19 or OGD/R + Gap26 treatment decreased percentage of CD40^+^CD11b^+^ microglia, while enhanced percentage of CD206^+^CD11b^+^ microglia; Further, OGD/R-ACM incubated with apyrase decreased percentage of CD40^+^CD11b^+^ microglial cells, while OGD/R + Gap19-ACM containing ATP-enhanced percentage of M1 subtype microglia; **b**(**1**-**3**), **c**(**1**-**2**) M1- or M2-related cytokines were evaluated by flow cytometry with CBA kits. We evaluated the statistical significance with ANOVA and Duncan’s multiple comparisons test. *p < 0.05, **p < 0.01, and ***p < 0.001

To determine microglial phenotypes induced by OGD/R injury, flow cytometry were applied to measure the expression of CD40 and CD206, which are markers for the M1 and M2 phenotypes, respectively. OGD/R injury increased the percentage of CD40^+^CD11b^+^ microglia and decreased the percentage of CD206^+^CD11b^+^ microglia. Effect of ACM on microglial polarization was also detected. ACM from the OGD/R group significantly increased percentage of CD40^+^CD11b^+^ microglia, while it decreased the percentage of CD206^+^CD11b^+^ microglia; OGD/R-Gap19 or OGD/R-Gap26 treatment decreased the percentage of CD40^+^CD11b^+^ microglia, while it enhanced the percentage of CD206^+^CD11b^+^ microglia. Further, OGD/R-ACM incubated with apyrase decreased the percentage of CD40^+^CD11b^+^ microglial cells, while OGD/R-Gap19-ACM containing ATP enhanced the percentage of M1 subtype of microglia (Fig. 10, a1–a2).

We also used flow cytometry with a CBA kit to measure concentrations of M1 phenotype-related pro-inflammatory cytokines (i.e., TNF-α, IFN-γ and IL-6) and M2 phenotype-related anti-inflammatory cytokines (i.e., IL-4 and IL-10) in cultured cell supernatants. The OGD/R group exhibited significantly increased pro-inflammatory cytokine concentrations, whereas the OGD/R + SalB group exhibited reduced pro-inflammatory cytokine concentrations and increased anti-inflammatory cytokine concentrations (*p* < 0.01). The ACM-treated microglia exhibited differential results. OGD/R-ACM treatment significantly induced elevation of concentration of TNF-α, IFN-γ, and IL-6 while it decreased concentration of IL-4 and IL-10. In comparison with OGD/R-ACM group, OGD/R-Gap19-ACM treatment reversed the effect. Similar results were obtained from OGD/R-Gap26-ACM and OGD/R + apyrase-ACM groups; OGD/R-Gap19 + ATP ACM application resulted in an apparent increase of these cytokines (*p <* 0.01) (Fig. 10, b(1-3), c(1-2).

### Effects of ACM on HT-22 neuronal cell lines after OGD/R injury

**Fig. 11:**
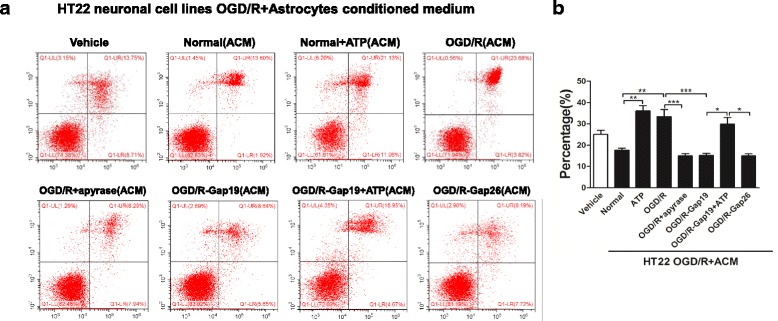
Effects of ACM on HT-22 neuronal cell lines subjected to OGD/R injury. **a**, **b** Cell apoptosis rates in HT-22 murine hippocampal cells, as measured by flow cytometry with an AnnexinV-FITC/PI Apoptosis Detection Kit. We evaluated the statistical significance with ANOVA and Duncan’s multiple comparisons test. *p < 0.05, **p < 0.01, and ***p < 0.001

To further explore hemichannel inhibitor-treated ACM’s effects on neuronal survival, HT-22 murine hippocampal neuronal cells were cultured and subjected to OGD for 12 h, then ACM were reperfused and cell viability was examined after a 48-h incubation period. Both ATP and OGD/R-ACM treatment increased HT-22 neurons apoptosis percentage compared with the vehicle group (*p* < 0.001). Further, HT-22 neurons treated with OGD/R + apyrase-ACM (15.38 ± 1.93%, *p <* 0.001), OGD/R-Gap19-ACM (16.12 ± 2.66%, *p <* 0.001), or OGD/R + Gap26-ACM (17.94 ± 3.74%, *p* < 0.05) attenuated cellular injury, while OGD/R-Gap19 + ATP-ACM enhanced HT-22 neuronal apoptosis (Fig. 11a, b).

### Effect of SalB and CBX on PKC and PKB kinases and Ser368- and Ser373-phosphorylated Cx43

**Fig. 12:**
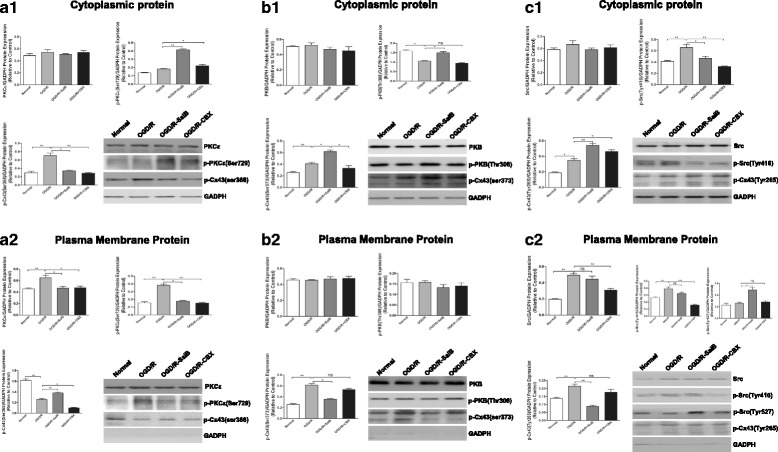
OGD/R injury with SalB or CBX application influenced astrocytic phosphorylated Cx43 and corresponding kinases. **a1**, **a2** OGD/R injury had no significant effect on cytoplasmic PKCε levels but significantly increased plasma membrane levels of PKCε and its Ser729-phosphorylated activated state. SalB and CBX reduced PKCε levels in the plasma membrane and increased them in the cytoplasm. Conversely, the OGD/R group’s Ser368-phosphorylated Cx43 levels were decreased in the plasma membrane and increased in the cytoplasm. SalB reversed these effects, but CBX reduced Ser368-phosphorylated Cx43 levels. **b1**, **b2** The OGD/R group exhibited decreased cytoplasmic levels of Thr308-phosphorylated PKB, though SalB reversed this effect. There were no significant changes in the plasma membrane levels. Furthermore, the OGD/R group exhibited elevated cytoplasmic and plasma membrane levels of Ser373-phosphorylated Cx43. SalB reduced the plasma membrane levels but further increased the cytoplasmic levels. CBX had little effect on the levels of Ser373-phosphorylated Cx43 and Thr308-phosphorylated PKB. **c1**, **c2** The OGD/R group exhibited elevated cytoplasmic and plasma membrane levels of Tyr416-phosphorylated Src. The OGD/R group also exhibited elevated plasma membrane levels of Src, which may be related to the elevated Tyr416-phosphorylated Src levels. SalB increased the plasma membrane levels of Src’s Tyr527-phosphorylated deactivated form but did not significantly affect plasma membrane levels of Tyr416-phosphorylated Src. CBX significantly reduced cytoplasmic and plasma membrane levels of Tyr416-phosphorylated Src. Furthermore, the OGD/R group exhibited increased cytoplasmic and plasma membrane levels of Tyr265-phosphorylated Cx43. SalB reversed this effect, but CBX achieved only non-significant reduction of the plasma membrane levels. We evaluated the statistical significance with ANOVA and Duncan’s multiple comparisons test. *p < 0.05, **p < 0.01, and ***p < 0.001

Phosphorylation of Cx43 at different sites controls the assembly, size, and turnover of gap junctions. Phosphorylation of Cx43 by PKB enhances gap junction assembly by increasing Cx43 trafficking to the plasma membrane [39], but Ser368-phosphorylation by PKC appears to mediate gap junction closure [40]. We therefore used western blotting to examine the levels of phosphorylated and dephosphorylated protein variants in the plasma membrane and cytoplasm of OGD/R, OGD/R + SalB, and OGD/R + CBX astrocytes (Fig. 12).

For OGD/R astrocytes, we found no obvious change in cytoplasmic levels of PKC epsilon type (PKCε) but significantly elevated plasma membrane levels of PKCε and its Ser729-phosphorylated activated state. SalB and CBX reduced the plasma membrane PKCε levels while increasing cytoplasmic PKCε levels. Conversely, the OGD/R-treated astrocytes’ Ser368-phosphorylated Cx43 levels were significantly decreased in the plasma membrane and significantly increased in the cytoplasm. SalB reversed these effects, but CBX inhibited both cytoplasmic and plasma membrane Ser368-phosphorylated Cx43 expression (Fig. 12, a1–2) (*p* < 0.05).

For OGD/R astrocytes, we found decreased cytoplasmic levels of Thr308-phosphorylated PKB, but SalB reversed this effect. We found no significant changes in the plasma membrane levels. Furthermore, the OGD/R astrocytes exhibited elevated cytoplasmic and plasma membrane levels of Ser373-phosphorylated Cx43. SalB reversed this effect in the plasma membrane but further elevated the cytoplasmic levels. CBX had little effect on the levels of Thr308-phosphorylated PKB or Ser373-phosphorylated Cx43 (Fig. 12, b1–2) (*p* < 0.05).

### Effects of SalB and CBX on Src kinases and Tyr265-phosphorylated Cx43 after OGD/R injury

SalB directly inhibits the activity of Src, which phosphorylates Cx43 at the Tyr265 site and thereby downregulates gap junction communication and promotes gap junction disassembly [47, 48]. We therefore investigated SalB’s effects on Src and Tyr265-phosphorylated Cx43 levels in the cytoplasm and plasma membrane.

Compared to the normal group, the OGD/R astrocytes expressed elevated cytoplasmic and plasma membrane levels of Src’s Tyr416-phosphorylated activated form and elevated plasma membrane Src levels, which may have been related to the elevated Tyr416-phosphorylated Src levels. SalB increased the plasma membrane expression of Src’s Tyr527-phosphorylated deactivated form but did not significantly affect plasma membrane expression of the Tyr416-phosphorylated form. CBX significantly reduced cytoplasmic and plasma membrane levels of Tyr416-phosphorylated Src. Furthermore, cytoplasmic and plasma membrane Tyr265-phosphorylated Cx43 levels were increased in the OGD/R group, but SalB significantly reversed this effect. CBX, however, induced only a slight, non-significant reversal of the elevated plasma membrane levels (Fig. 12, c1–2) (*p <* 0.05).

## Discussion

### SalB and CBX modulated astrocytic Cx43 expression, hemichannel permeability, and gap junction communication after OGD/R injury

In this study, we measured subcellular compartment-specific Cx43 protein levels in astrocytes. SalB and CBX similarly reversed OGD/R injury-induced internalization of plasma membrane Cx43 but did not significantly change total cellular Cx43 levels. These results are consistent with our previous observation that ischemic injuries induced cytoplasmic internalization of Cx43 in rat astrocytes but did not change total Cx43 levels [77]. However, some studies also reported increased Cx43 expression post-ischemic [78–80]. Nakase and colleagues were first to investigate Cx43 expression levels in the human brain, and they found increased Cx43 levels after long-term ischemia [79]. Another study by Xie et al. revealed that inhibiting Cx43 upregulation significantly increased pyramidal neuron survival and alleviated cognitive impairments after middle cerebral artery occlusion [12]. We speculate that the discrepancies may arise from differences between in vivo and in vitro models, as elevated Cx43 expression in vivo may result from astrogliosis, whereas in vitro studies usually focus on Cx43 protein levels per cell. In 2010, Orellana and colleagues demonstrated that hypoxia/reoxygenation causes a transient increase in astroglial surface Cx43 protein levels [80], which has been assumed that surface Cx43 is in the form of hemichannels [81]. Hence, the authors indicated that the increased hemichannel-related Cx43 proteins could account for the increased Cx43 hemichannel activity, which were also in accordance with works by Retama and colleagues in 2006 [82]. Here, we applied a commercial plasma membrane protein isolation kit from Invent Biotechnologies, as guided in the protocols; plasma membrane subtraction was separated from a mixture of nuclei, cytosol, and organelles by subsequent differential centrifugation and density centrifugation. The methods for plasma membrane extraction employed here showed no selectivity for gap junctional or hemichannel-related Cx43 proteins; in other words, the methods used in our research allowed collection of both hemichannel- and gap junction channel-related Cx43 proteins. In the current study, we found that, in OGD/R groups, plasma membrane Cx43 protein levels were apparently downregulated, while cytoplasma Cx43 protein levels were upregulated, compared with that in normal cultured astrocytes (Fig. 1). It has been proposed that under control situations, only about 15% of the total astrocytic Cx43 proteins expressed was in hemichannels [82]; thus, we speculated that the decreased plasma membrane Cx43 proteins levels were mainly those Cx43 proteins composing gap junctional channels, which were internalized into cytoplasma for further degradation after OGD/R stimulation.

In addition to Cx43 protein levels, studies have also investigated astrocytic Cx43 hemichannel activity and GJIC coupling. Here, we found that subjecting astrocytes to OGD/R injury prominently increased their ethidium uptake ability and supernatant ATP concentrations but decreased astrocytic dye coupling degree. SalB or CBX treatment both achieved significant attenuation of the effects on ethidium uptake and ATP release. Furthermore, SalB treatment enhanced astrocytic cellular dye transfer, while CBX application showed inhibited effects (Fig. 2).

Hemichannels release relevant quantities of signaling molecules (e.g., ATP, glutamate, NAD^+^ and PGE2) to the extracellular milieu [83]. In vitro ischemia-like conditions enhance hemichannel activity in astrocytes and many other cell types [7]. Studies have provided strong evidence that deleterious hemichannels open after cerebral ischemia [84, 85]. In the current study, we performed dye uptake by astrocytes with EtBr incubation and bioluminescence for determination of eATP concentration, both were indicators for hemichannel activity, and found that increased astrocytic hemichannel opened under OGD/R injury, in accordance with those previous studies. However, it should be noticed that both connexin and pannexin expressed on astrocytes contribute to hemichannels [7]. Here, we applied CBX, blocker for both Cx and pannexin channels [86], which showed inhibition for astrocytic hemichannel activity. Further study specially target Panx1 with ^10^Panx1 may distinguish and investigate the possible contribution of Px1 hemichannels and Cx43 hemichannels [7]. In particular, Orellana et al. found no significantly increased dye uptake in Cx43-deficient astrocytes after hypoxia. In addition, Cx43 mimetic peptide prevented hypoxia induced dye uptake by hemichannel in astrocytes, but not by pannexin hemichannel blockers [80]. Iwabuchi and Kawahara have proposed a complex negative feedback loop for pannexin hemichannels, whereby released ATP acts via P2X7 receptors to induce Pan1 hemichannel closure [87].

Gap junction channels have evident physiological significance in morphogenesis, development, and tissue synchronization, but two opposing hypotheses exist in regard to their role in cell death. The transfer of caspase-derived apoptotic peptides through gap junction channels supports a “bystander” hypothesis, as studies showing that non-selective gap junction blockers, such as octanol [88] and CBX [89, 90], provide protection in models of brain ischemia. In contrast, a “good Samaritan” role is supported by studies showing that Cx43 gene knockout is associated with larger stroke lesions, amplified apoptosis, and inflammation [91, 92]. Furthermore, post-injury gap junction channel inhibition correlates with glutamate cytotoxicity and neuronal injury aggravation [93, 94]. Our findings seem to support the “good Samaritan” hypothesis, but there may be a balance between the “bystander” and “good Samaritan” hypotheses. The known discrepancies probably arise from the use of non-selective gap junction blockers that also inhibit hemichannels.

In conclusion, we observed opened hemichannels, weakened GJIC, and Cx43 internalization in astrocytes after OGD/R injury. Both CBX and SalB inhibited Cx43 redistribution. CBX suppressed the opening of hemichannels and gap junctions; SalB enhanced cell communication while reducing hemichannel openings.

### Effects of ACM from SalB- and CBX-treated astrocytes on microglial activation after OGD/R injury

Inflammatory responses contribute to secondary neuronal damage, which substantially affects acute ischemic injuries. After ischemia, newly activated microglia produce both detrimental and neuroprotective mediators, with the balance between them determining the injured neurons’ fates. Activated microglia can exhibit either the classic M1 pattern, in which they secrete pro-inflammatory cytokines and exacerbate neuronal injuries, or the alternative M2 pattern, in which they promote reparative anti-inflammatory responses [27]. Several receptors expressed on microglia recognize specific ligands, including heat shock proteins, ATP, and nucleic acids [95, 96]. Ischemia-induced neuronal death results in ATP release and microglial activation via P2 receptors. This corresponds with significant post-ischemic elevation of microglial P2X4 and P2X7 receptor expression [97, 98]. Although many factors mediate the migration of activated microglia to the injured region, ATP is among the most important mediators [99]. Extracellular ATP induces endogenous ATP release from microglia, which attracts distant microglia to the injury site [123].

ATP release through astrocytic hemichannels establishes an ATP gradient that is a critical trigger for microglial responses. In 2005, Davalos et al. showed that local ATP injections mimicked traumatic brain injuries and induced microglial activation, which was inhibited by connexin channel blockers [100]. This indicates that extracellular ATP released from damaged tissues and surrounding astrocytes mediates a rapid microglial injury response. Furthermore, Huang et al. showed that Cx43 knockout mice exhibited diminished areas of post-traumatic ATP release, suppression of astrogliosis and microgliosis, and less tissue loss following spinal cord injuries [101]. Similarly, another study showed that partial deletion of astrocytic Cx43 expression similarly reduced pro-inflammatory cytokine levels after systemic lipopolysaccharide injections [26]. Moreover, partial Cx43 deletion inhibits microglial activation in mice, and hemichannel modulators such as Cx43 mimetic peptide [24] and Cx43 antisense oligodeoxynucleotide [102] effectively inhibit post-spinal cord injury inflammation. These results suggest that connexin hemichannels act as a “switch” for the inflammasome signaling cascade by contributing extracellular ATP both during and after an injury. Here, we found that SalB attenuated OGD/R injury-induced microglial activation, including the morphology changes, M1/M2 polarization, and release of pro-inflammatory or anti-inflammatory cytokines. Furthermore, when applied to microglia, OGD/R + SalB-ACM and OGD/R + CBX-ACM induced weaker microglial inflammatory reactions than OGD/R-ACM did, which is consistent with the results of the previous studies (Figs. 4 and 5).

In particular, it should be noticed that neither SalB nor CBX was a specific Cx43 hemichannel or gap junctional blocker [49, 54, 103]. Hence, we further applied specific Cx43 hemichannel blocker-Gap 19 to explore the role of astrocytic Cx43 hemichannel during OGD/R injury. Results indicated that Gap 19 application significantly blocked OGD/R-induced Cx43 hemichannel opening and ATP release. Furthermore, OGD/R-ACM promoted microglial activation and HT-22 neuronal apoptosis, while after incubation with apyrase for 30 min, OGD/R + apyrase-ACM attenuated microglial activation and HT-22 neuronal injury. Also, OGD/R-Gap19-ACM obviously attenuated microglial activation, while addition of ATP in OGD/R-Gap19-ACM enhanced microglial activation and HT-22 neuronal damage. Another gap junctional inhibitor Gap26 showed similar results to those of Gap19 (Figs. 8, 9, 10, and 11).

We conclude that OGD/R injuries induce astrocytic Cx43 hemichannel opening and thus cause substantial ATP release, which plays an important role in microglial activation and HT-22 neuronal survival during OGD/R injury process. SalB and CBX may exert their protective effects by reducing ATP release; further study using Cx43 mimetic peptide Gap19 established the critical role of astrocytic Cx43 hemichannel and the secondary released ATP during OGD/R injury-induced neuroinflammatory responses.

### Effects of MCM on astrocytic hemichannels and gap junctions after OGD/R injury

Previous studies showed that incubating astrocytes with pro-inflammatory cytokines or a high proportion of microglia caused reduced Cx43 expression and dye coupling accompanied with extensive microglial activation. Adding the anti-inflammatory mediator transforming growth factor β1 reverses the microglial activation and restores functional coupling [28, 30]. We cannot exclude the possibility that cytokines directly affect astrocytic properties like Cx43 expression, especially given the evidence that the pro-inflammatory cytokine IL-1β directly affects astrocytic gap junctions [104, 105]. Similarly, it has been reported that amyloid β (Aβ) induces microglial activation and thereby influences astrocytic gap junctions [106] and that CB treatment prevented Aβ-induced astrocytic hemichannel activation [107].

In our study, treating astrocytes with OGD/R-MCM induced a prominent increase in ethidium uptake but reduced cell coupling, but using OGD/R + SalB-MCM reversed these effects (Fig. 6). The mechanism remains unclear, though the functional interference may involve phosphorylation, since Cx43 function is quite sensitive to various kinases and phosphatases, including MAPK. For instance, brain slice studies have shown that ischemia, which upregulates the expression of cytokines such as IL-1β and TNF-α, induces Cx43 dephosphorylation [108]. Furthermore, cytokines affect other astrocytic properties. For example, the pro-inflammatory cytokine TNF-α activates PKC, which causes depolarization of astrocytes [105]. Further research is necessary to clarify the mechanisms.

In conclusion, our findings indicate that activated microglia and their pro-inflammatory cytokine secretions differentially regulate astrocytic gap junctions and hemichannel activity, which may in turn aggravate ATP release from opened hemichannels and thus form a vicious circle after OGD/R injury.

### Effects of SalB and CBX on Src, PKC, and PKB and the corresponding Cx43 regulatory sites in astrocytes after OGD/R injury

Phosphorylation of Cx43’s C-terminal domain regulates GJIC. This domain is phosphorylated at over a dozen residues [37–40]. Many kinases phosphorylate Cx43, and the predominant effect is a decrease in GJIC [41]. In the ischemic penumbra, significant changes happen in the states of numerous signaling pathways involving those protein kinases, including MAPK family members, PKB and PKC kinases [43–47]. In our study, we assessed protein expression in OGD/R-injured astrocytes and found that Ser368-phosphorylated Cx43 levels were decreased in the plasma membrane but increased in the cytoplasm. Furthermore, PKC, which phosphorylates the Cx43’s Ser368 site, was significantly upregulated and activated in the plasma membrane. Our results were similar to those of a previous immunohistochemistry study that showed ischemia-induced dephosphorylation of astrocytic Cx43 [109]. However, it remains unclear how both Cx43 dephosphorylation and PKC activation occur during OGD/R injury, as under normal conditions, Ser368-phosphorylated Cx43 levels remain whereas no PKC activation is observed. OGD/R injury may induce some other unknown factors. Research has showed that uncoupling of Cx43-based GJIC was more a cause than a consequence of Cx43 dephosphorylation because post-hypoxic decreases in astrocytic coupling occurred before Cx43 dephosphorylation [41]. In cultured astrocytes exposed to hypoxia, Cx43 dephosphorylation occurs in conjunction with reduced GJIC [110, 111]. In our study, we found that SalB inhibited PKC activation and upregulated Ser368-phosphorylation of Cx43, which may be related to enhanced astrocytic coupling. However, CBX inhibited PKC activation and reduced Ser368-phosphorylation of Cx43, which indicates that Cx43 or Cx43-related GJIC may also exert regulatory effects on PKC activity.

Ser373-phosphorylated Cx43 is also associated with dramatically increased gap junction size and gap junctional communication [114]. Akt induces Ser373 phosphorylation of Cx43 [101], and inhibiting Akt causes gap junction losses [39]. Here, in the OGD/R group astrocytes, we found increased levels of Ser373-phosphorylated Cx43 in both the plasma membrane and cytoplasm and reduced cytoplasmic levels of PKB’s Thr308-phosphorylated activated form. Solan and Lampe explored post-injury gap junctional upregulation and turnover in a model of wound healing and found that under conditions of injury or growth factor treatment, the first step is characterized by increased gap junction size and gap junctional communication and Akt activation [40]. Besides, Ser373-phosphorylation of Cx43 occurs within 5–15 min after injury [113]. They suggested that this initial step served to deplete the plasma membrane of non-junctional Cx43 by rapidly incorporating it into gap junctions and efficiently internalizing it. In our study, we detected these proteins after a 48-h reperfusion period, which may account for the opposite effects on PKB activity and Ser373-phosphorylated Cx43 levels, because after 48 h, most of the astrocytic Cx43 would already have been internalized. Further studies are necessary to investigate these changes in the early periods after OGD/R injuries. An interesting direction for such studies was indicated by Bejarano et al. [114], in which they showed that connexins modulate autophagosome biogenesis and observed internalization of connexin-autophagy protein complexes. Furthermore, novel electron microscopy techniques have also been used to show localization of phosphorylated Cx43 in mouse ovarian follicles [10]. Furthermore, we found that SalB reduced plasma membrane levels of Ser373-phosphorylated Cx43 but increased cytoplasmic Thr308-phosphorylated PKB levels, whereas CBX exerted no such effects. Besides its function in phosphorylating Cx43, PKB is involved in myriad cellular processes including cell survival, metabolism, and protein synthesis [116]. SalB-induced Thr308- phosphorylation of PKB may also provide protection.

Src has long been known to downregulate gap junction communication and cause gap junction disassembly by phosphorylating Cx43 [117, 118]. Src directly phosphorylates Cx43’s Tyr247 and Tyr265 residues [119, 120]. In this study, we showed that OGD/R injury significantly activated Src, as indicated by the upregulation of cytoplasmic and plasma membrane levels of Tyr416-phosphorylated Src. Furthermore, the OGD/R group also exhibited increased plasma membrane levels of Tyr265-phosphorylated Cx43. This is consistent with previous studies [41, 42]. Interestingly, in a wound healing model in which Akt phosphorylated Cx43 within 5–15 min of the injury, Src exerted its function within 30 min and continued doing so for 24 h or longer. This was accompanied by rapid downregulation of gap junctional communication and gap junctional internalization, which is critical to later steps in effective wound healing [121]. Similar phenomena are also observed in ischemic pathologies. For example, Li et al. found that chemical ischemia/hypoxia induced marked astrocytic Cx43 dephosphorylation, and the “dephosphorylated” form of connexin-43 was immunoprecipitated by a phosphotyrosine antibody [41], suggesting tyrosine phosphorylation of connexin-43 by Src. Furthermore, inhibiting Cx43 dephosphorylation blocked Src-Cx43 interactions. Naitoh et al. showed that in isolated rat hearts, PKCε was co-immunoprecipitated with Cx43 in the non-ischemic myocardium and that the levels of both increased after the onset of ischemia [42]. Cx43-Src complexes were detected 35 min after ischemia but not under the baseline condition or at 10 min after ischemia. We therefore conjecture that after the 48-h reperfusion period in our study, Src had been activated, Cx43’s Tyr265 site had been phosphorylated, and large-scale Cx43 internalization was underway.

Recently, Pan and co-workers showed that SalB directly inhibited Src activity [57]. We found that SalB increased astrocytic plasma membrane levels of Src’s Tyr527-phosphorylated deactivated form but did not significantly decrease plasma membrane levels of Tyr416-phosphorylated Src, which may be due to incomplete dephosphorylation [122]. However, SalB did decrease cytoplasmic levels of Tyr416-phosphorylated Src. As for Tyr265-phosphorylated Cx43, SalB decreased plasma membrane levels but increased cytoplasmic levels. These results indicate that SalB inhibited Src and reduced Tyr265-phosphorylated Cx43 levels. Combined with our observations that SalB decreased Ser373-phosphorylated Cx43 levels and increased Ser368-phosphorylated Cx43 levels in the plasma membrane, we conclude that SalB-induced Src inhibition may promote Ser368-phosphorylation of Cx43, which is associated with Cx43-related GJIC under normal conditions.

CBX is a semisynthetic derivative of glycyrrhetinic acid [124]. It has been demonstrated that CBX produced inhibition of the both hemichannel and gap junctional intercellular communication [125, 126]. In the current study, 10 μM of CBX was selected based on MTT-viability tests for astrocytes implanted for OGD/R injury, as shown in Additional file 1: Figure S1B. Further, WB analysis for various phosphorylated Cx43 proteins and related protein kinases showed that CBX treatment induced obviously downregulation of p-Cx43(Ser368), accompanied by decreased p-PKC(Ser729) protein levels in plasma membrane, while showing no significantly regulation for p-Cx43(Tyr265) and p-Cx43(Ser373). Besides, CBX treatment inhibited plasma membrane’s Src kinases activity, with markedly decreased p-Src(Tyr416) protein levels. Here, several issues need to be mentioned. First, although it has been widely used, the potential inhibitory mechanism of CBX is still not completely understood until now. Verselis and Srinivas indicated that it may be that these reagents work through protein internalization or turnover, or perhaps an indirect mechanism involving binding to cytoplasmic intermediate molecules [127, 128]. Thus, the results in our research prompt further investigation for CBX’s potential action targets. Second, studies have found that CBX with different concentrations of 10, 20, and 100 μM (10 min) caused a dose-dependent attenuation of dye coupling by − 71 ± 4%, − 85 ± 4%, and − 92 ± 2%, respectively, as assessed by the scrape-loading technique [129]. Another study showed that, compared with untreated cells, 50 μmol/L CBX reduced the strength of homocellular coupling between astrocytes by about 70% using fluorescence recovery after photobleaching (FRAP) method [90]. Study also showed that EtBr uptake is reduced by Cx knockdown (50.4% inhibition) and CBX treatment (40.8%; 100 μM) [70]. In our research, dye uptake is reduced by about 80% after CBX treatment, compared with the OGD/R groups, while for dye coupling, no obvious changes observed in those two groups, for OGD/R in the current research also induced attenuation of gap junction communication among astrocytes. Conclusively, the inhibitory effects of CBX on astrocytic hemichannels and GJ were concentration dependent, while different methods for evaluating the channels’ permeabilities probably account for inconsistency of results obtained. In summary, further studies still need to focus on the concentration related-regulation for Cx43 hemichannels and gap junctional permeability. Also, the methods used for evaluating hemichannel activity or gap junction conduction need to be taken into consideration. Otherwise, the mechanism of Panx1 channel inhibition of carbenoxolone has been elucidated and it seems that a mutation in the first extracellular loop reverses its action polarity [130].

Besides, we assumed that Cx43 may be one of the most important targets of SalB, for we found accidentally that, following CBX pre-treatment of astrocytes for 30 min, SalB exerted more deleterious effect than protection for astrocytes suffering OGD/R injury; in addition, Gap19 or Gap26 exerted similarly results. On the other hand, Gap 19, Gap26, or CBX treatment following SalB pre-treatment showed similar protection with the effect of single-drug application for astrocytes exerted to OGD/R injury (Additional file 1: Figure S1C). One possible explanation for this phenomenon may be derived from the mechanism of drug resistance—cell adhesion-mediated drug resistance (CAM-DR). Fulda et al. reported that glioblastoma multiform (GBM) cells are able to employ CAM-DR by forming spheres via cell–cell interactions. Intriguingly, when inhibiting cell–cell interactions by inhibition of gap junctions through chemical inhibition with carbenoxolone or connexin-mimicking Gap27, GBM cells were sensitized to drug-induced apoptosis. Nevertheless, further research to explore the real truth is needed.

### Study limitations

This study has some limitations. First, application of selective pannexin hemichannel blockers such as ^10^Panx1 could have provided more precise observations about hemichannel activity. Furthermore, it should be noted that hemichannels release not just ATP but also glutamate and other molecules. The roles of other connexins such as Cx30 should also be considered, though Cx43 is the main astrocytic connexin. Second, we only examined a single timepoint, but a comparison of our results to previously published ones suggests that a timepoint has an important effect on Cx43 distributions. Examining earlier timepoints and performing dynamic and continuous observations may provide more comprehensive results. In vivo studies may also provide further elucidation.

## Conclusions

**Fig. 13:**
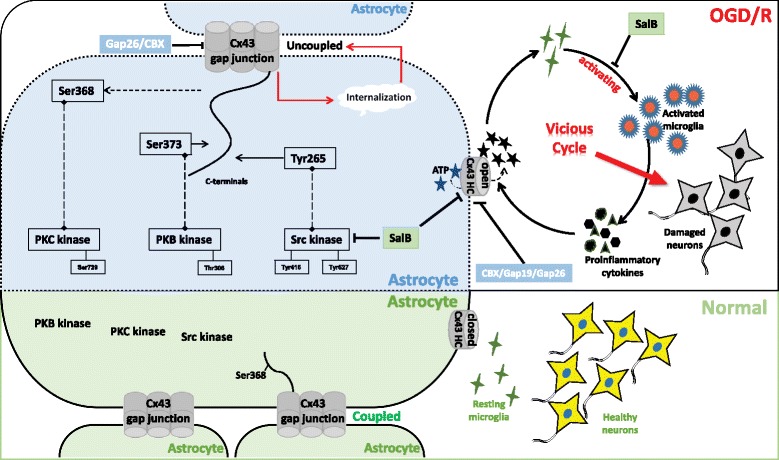
Schematic showing potential roles of astrocytic Cx43, hemichannels, and GJIC during OGD/R injury. Under normal conditions, astrocytic Cx43 is expressed in the plasma membrane and assembled into hemichannels that are normally closed. Hemichannel-hemichannel interactions induce the formation of GJIC between adjacent astrocytes, which permits the exchange of ions and small molecules; also, plasma membrane’s Cx43 was phosphorylated at Ser368 site. In such circumstances, astrocytes, together with those resting microglia, function as a supportive assistant for healthy neurons. OGD/R injury caused abnormal hemichannel opening and consequent substantial astrocytic ATP release. It also induced microglial activation with a predominance of the pro-inflammatory cytokine-releasing M1 subtype. Extracellular ATP induced further microglial activation and pro-inflammatory cytokine release, and these pro-inflammatory cytokines induced further opening of astrocytic hemichannels. SalB reversed these effects and thus provided protection against OGD/R injury. This suggests the existence of a vicious cycle in which astrocytic hemichannel opening and pro-inflammatory microglial activation reinforce each other following OGD/R injury. This vicious cycle may account for secondary injury and extended damage after OGD/R injury; OGD/R injury caused gap junction internalization, which may account for the astrocytic uncoupling events. It also decreased plasma membrane levels of Ser368-phosphorylated Cx43 while increasing plasma membrane levels of Ser373-phosphorylated Cx43, Ser265-phosphorylated Cx43, and Src’s Tyr416-phosphorylated activated form. The activated Src may well have phosphorylated Cx43 at Tyr265 and further induced gap junction internalization or autophagy. SalB directly inhibits Src, which may allow it to exert protective effects by attenuating Cx43 internalization. CBX, a non-selective hemichannel and GJIC inhibitor, did not apparently affect Cx43 phosphorylation, but it inhibited PKC and Src activity

This study provides two major new findings (Fig. 13). The first is that OGD/R injury induced redistribution and apparent internalization of astrocytic Cx43, with abnormal hemichannel opening, ATP release, and reduced GJIC coupling. Furthermore, ATP released from Cx43 hemichannels induced microglial activation with the M1 subtype predominating. Based on these findings, we further explored the interrelationship between astrocytes and microglia with cell-conditioned media. The ACM contained higher ATP concentration and increased microglial activation and secondary release of pro-inflammatory cytokines, whereas the MCM induced astrocytic hemichannel opening while reducing GJIC coupling. SalB provided neuroprotection by reversing the abnormal opening of astrocytic hemichannels, reducing ATP release, and switching the activated microglial subtype from M1 to M2. Our results suggest the existence of a vicious cycle between astrocytic hemichannel opening and pro-inflammatory microglial secretions after OGD/R injury, such that ATP release induces pro-inflammatory cytokine secretion that induces further ATP release. The vicious cycle may account for secondary injuries and extended damage after OGD/R injury. Our second major finding concerns multisite phosphorylation of Cx43’s C-terminal region and the corresponding kinases. We found that our OGD/R protocol internalized most Cx43 variants, but the plasma membrane levels of both Ser265-phosphorylated Cx43 and Tyr416-phosphorylated Src were significantly increased. We conclude that activated Src probably phosphorylates Cx43 at the Tyr265 site and further induces gap junction internalization. SalB may exert protective effects by inhibiting Src and attenuating Cx43 internalization. CBX is a non-selective hemichannel and GJIC inhibitor. CBX treatment induced obviously downregulation of p-Cx43(Ser368) and p-PKC(Ser729) protein levels in plasma membrane, which may prompt us to further study potential action target of CBX.

## Additional file

Additional file 1: **Figure S1**. Analysis of purity of primary cultured astrocytes or microglia. Primary glial cells were prepared, astrocytes and microglial cells were prepared and purified. (A1) Cells were stained with anti-CD11b-FITC antibody and detected with flow cytometry. The staining showed that < 0.1% of the cultured cells were microglia; (A2) Immunofluorescent staining revealed astrocytes stained with GFAP (green). Nuclei were stained with DAPI (blue). The GFAP staining showed that > 98% of the cultured cells were astrocytes. For microglial cells separated from the mix culture, both flow cytometry analysis and immunofluorescent staining showed that > 99% of the cultured cells were microglia in (B1-B2). Scale bar = 50 μm. Figure S2. MTT assay for cell viability of astrocytes undergone OGD/R injury. Primary astrocytes were prepared from newborn mice and subjected to OGD/R injury. (A) MTT assay to measure cell viability in astrocytes after treatment with SalB at 5 to 100 μg/mL concentrations. Con: control; (B) MTT assay to measure cell viability in astrocytes after treatment with CBX at 10 to 5000 μM concentrations. Con: control; (C) MTT assay to measure cell viability in astrocytes after treatment with CBX at 10 μM, SalB at 20 μg/mL, Gap19 at 100 μM, Gap26 at 100 μM; Also, Gap19, Gap26 or CBX pretreatment followed by SalB incubation and SalB pretreatment for 30 min followed by Gap19, Gap26 or CBX incubation with the above indicated concentrations; All error bars:±SEM. We evaluated the statistical significance with ANOVA and Duncan’s multiple comparisons test. *p < 0.05, **p < 0.01, and ***p < 0.001. Figure S3. Standard curve for ATP detection. ATP levels in conditioned medium were determined. The fluorescence levels from five serial ATP dilutions—0, 10, 30, 60, 100, 300, and 1000 nM are shown. Figure S4 (A-B) Western blotting were performed to evaluate the M2 marker arginase-1. Arginase-1 protein expression was decreased in the OGD/R group’s activated microglia, but SalB reversed this effect; (C-D) Arginase-1 expression was decreased in OGD/R-ACM-treated microglia while increased in microglia treated with OGD/R-SalB-ACM or OGD/R-CBX-ACM. We evaluated the statistical significance with ANOVA and Duncan’s multiple comparisons test. *p < 0.05, **p < 0.01, and ***p < 0.001. (PPTX 11400 kb)

## Abbreviations

ACM: Astrocyte-conditioned medium
ATP: Adenosine triphosphate
CBA: Cytometric bead array
CBX: Carbenoxolone
CK1: Casein kinase 1
CNS: Central nervous system
Cx43: Connexin-43
DMEM: Dulbecco’s modified Eagle’s medium
EtBr: Ethidium bromide
FBS: Fetal bovine serum
FRAP: Fluorescence recovery after photobleaching
GFAP: Glial fibrillary acidic protein
GJIC: Gap junction intercellular communication
I/R: Ischemia/reperfusion
IL-1β: Interleukin-1β
MAPK: Mitogen-activated protein kinase
MEM: Microglia-conditioned medium
PFA: Paraformaldehyde
PKB: Protein kinase B
PKC: Protein kinase C
PVDF: Polyvinylidene fluoride
SalB: Salvianolic acid B
TNF-α: Tumor necrosis factor-α
