# Supplementary material for: Roles of astrocytic connexin-43, hemichannels, and gap junctions in oxygen-glucose deprivation/reperfusion injury induced neuroinflammation and the possible regulatory mechanisms of salvianolic acid B and carbenoxolone
Source: J Neuroinflammation. 2018 Mar 27;15:97. doi: 10.1186/s12974-018-1127-3 (PMC5872583; doi:10.1186/s12974-018-1127-3)
Supplement: Supplementary file 1 — Figure S1. Analysis of purity of primary cultured astrocytes or microglia. Primary glial cells were prepared, astrocytes and microglial cells were prepared and purified. (A1) Cells were stained with anti-CD11b-FITC antibody and detected with flow cytometry. The staining showed that < 0.1% of the cultured cells were microglia; (A2) Immunofluorescent staining revealed astrocytes stained with GFAP (green). Nuclei were stained with DAPI (blue). The GFAP staining showed that > 98% of the cultured cells were astrocytes. For microglial cells separated from the mix culture, both flow cytometry analysis and immunofluorescent staining showed that > 99% of the cultured cells were microglia in (B1-B2). Scale bar = 50 μm. Figure S2. MTT assay for cell viability of astrocytes undergone OGD/R injury. Primary astrocytes were prepared from newborn mice and subjected to OGD/R injury. (A) MTT assay to measure cell viability in astrocytes after treatment with SalB at 5 to 100 μg/mL concentrations. Con: control; (B) MTT assay to measure cell viability in astrocytes after treatment with CBX at 10 to 5000 μM concentrations. Con: control; (C) MTT assay to measure cell viability in astrocytes after treatment with CBX at 10 μM, SalB at 20 μg/mL, Gap19 at 100 μM, Gap26 at 100 μM; Also, Gap19, Gap26 or CBX pretreatment followed by SalB incubation and SalB pretreatment for 30 min followed by Gap19, Gap26 or CBX incubation with the above indicated concentrations; All error bars:±SEM. We evaluated the statistical significance with ANOVA and Duncan’s multiple comparisons test. *p < 0.05, **p < 0.01, and ***p < 0.001. Figure S3. Standard curve for ATP detection. ATP levels in conditioned medium were determined. The fluorescence levels from five serial ATP dilutions—0, 10, 30, 60, 100, 300, and 1000 nM are shown. Figure S4 (A-B) Western blotting were performed to evaluate the M2 marker arginase-1. Arginase-1 protein expression was decreased in the OGD/R group’s activated microglia, but SalB reversed [file 12974_2018_1127_MOESM1_ESM.pptx]

## Slide 1
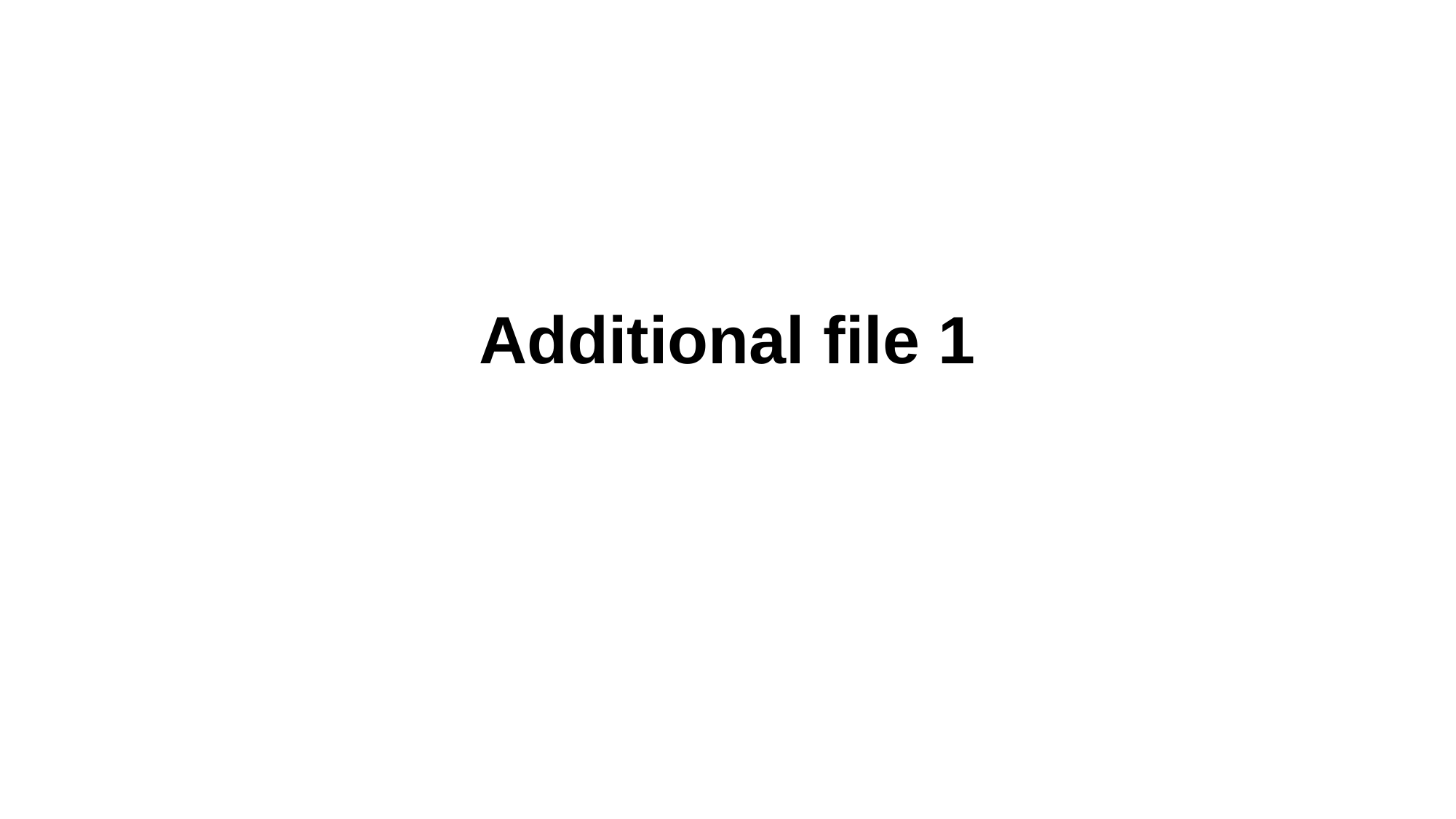

Additional file 1

## Slide 2
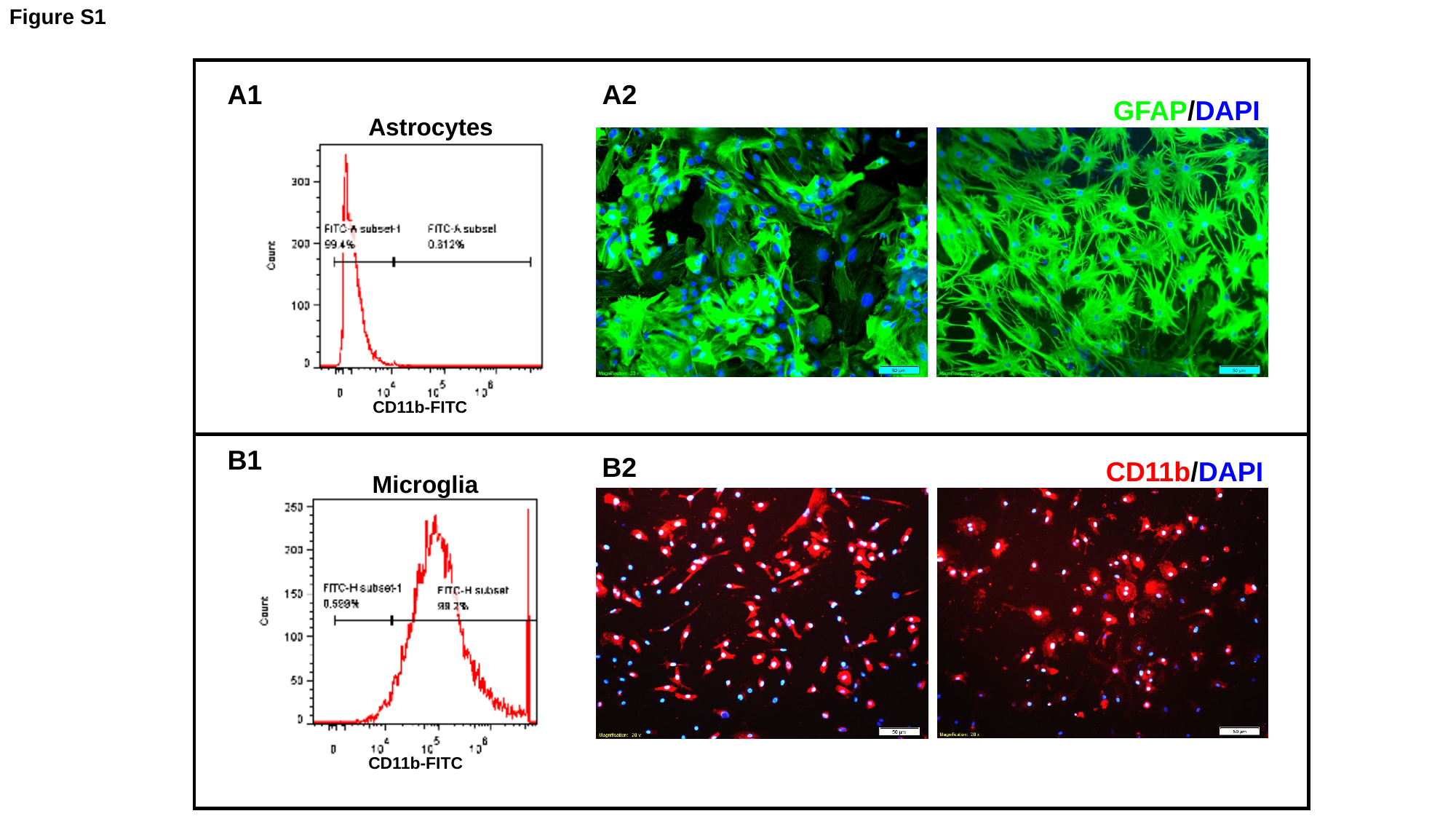

Figure S1
A1
A2
GFAP/DAPI
Astrocytes
CD11b-FITC
B1
B2
CD11b/DAPI
Microglia
CD11b-FITC

## Slide 3
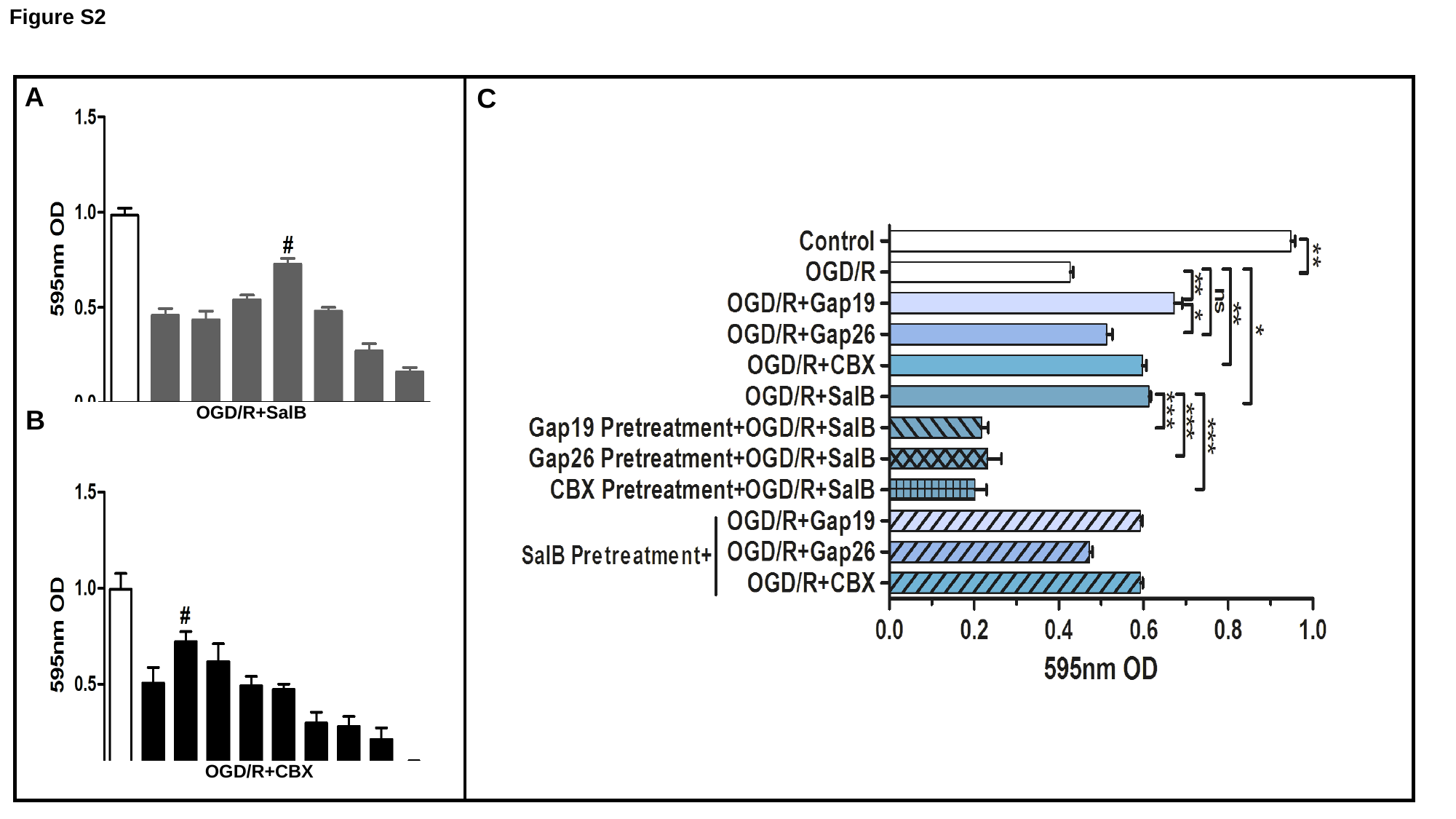

Figure S2
A
C
OGD/R+SalB
B
OGD/R+CBX

## Slide 4
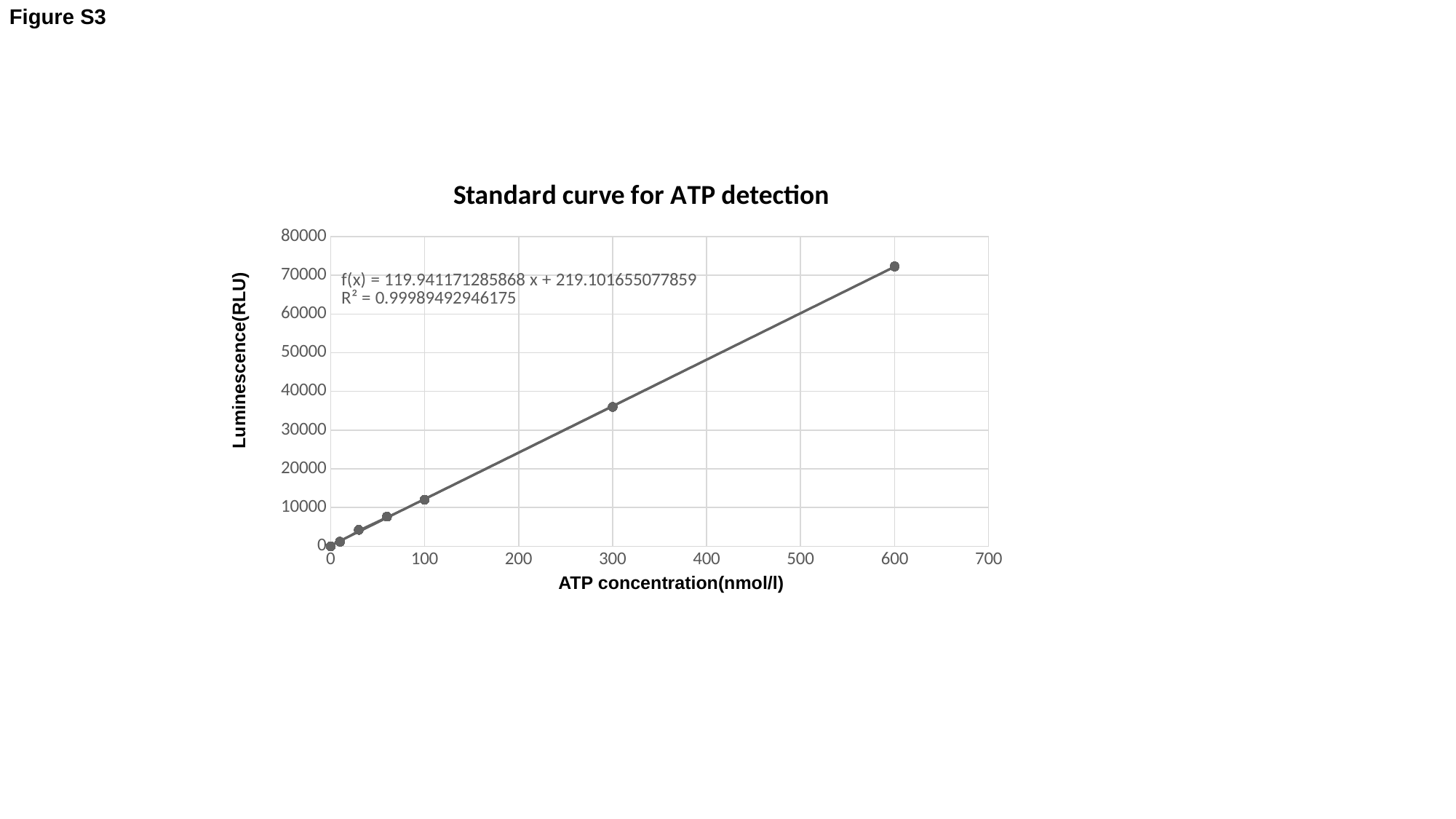

Figure S3
### Chart: Standard curve for ATP detection
| Category | |
|---|---|Luminescence(RLU)
ATP concentration(nmol/l)

## Slide 5
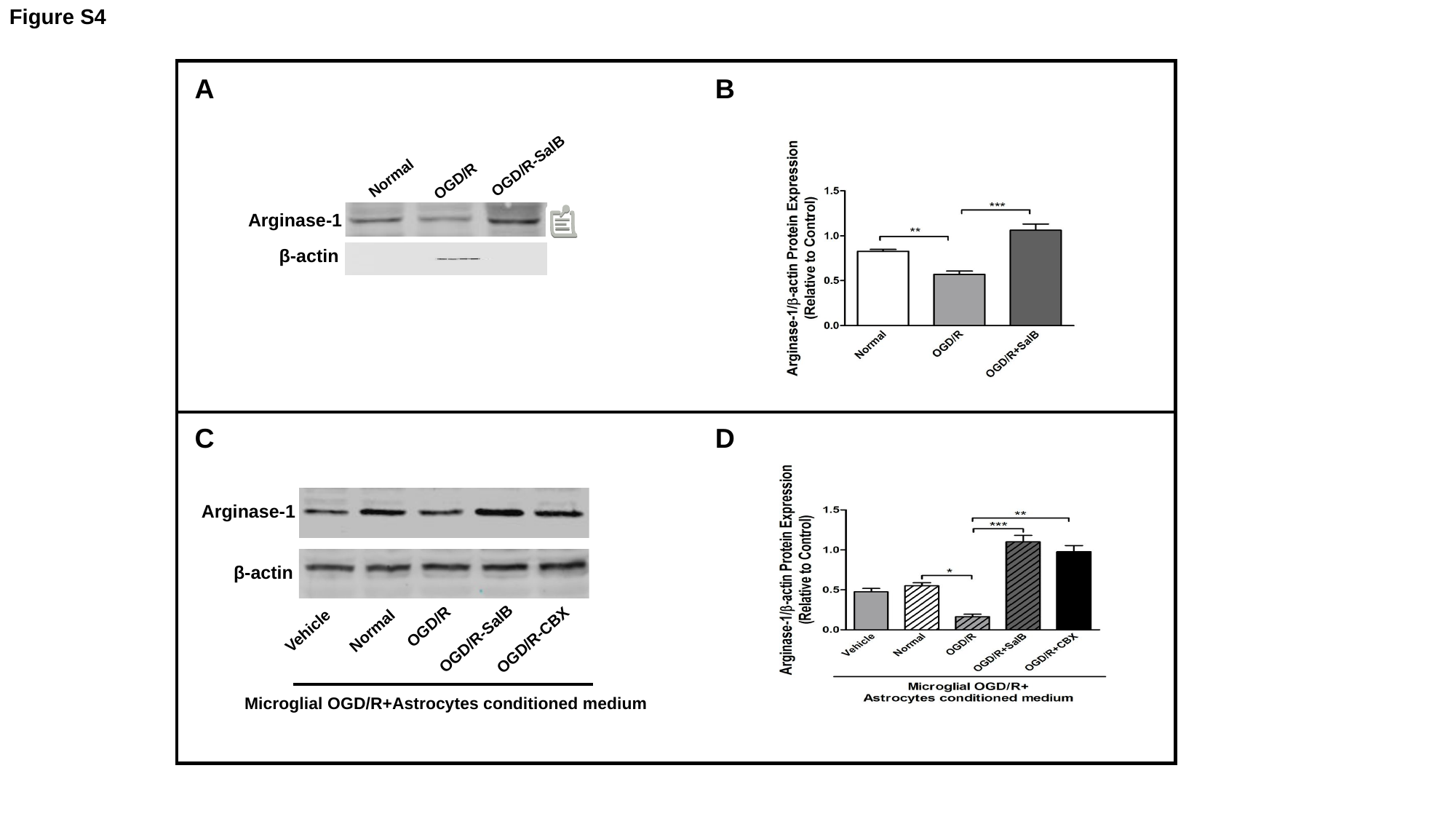

Figure S4
A
B
OGD/R-SalB
Normal
 OGD/R
Arginase-1
β-actin
C
D
Arginase-1
β-actin
OGD/R
Normal
Vehicle
OGD/R-CBX
OGD/R-SalB
Microglial OGD/R+Astrocytes conditioned medium
